# Root avoidance of toxic metals requires the GeBP‐LIKE 4 transcription factor in *Arabidopsis thaliana*

**DOI:** 10.1111/nph.14242

**Published:** 2016-10-21

**Authors:** Deepa Khare, Nobukata Mitsuda, Seungchul Lee, Won‐Yong Song, Daehee Hwang, Masaru Ohme‐Takagi, Enrico Martinoia, Youngsook Lee, Jae‐Ung Hwang

**Affiliations:** ^1^ Department of Life SciencePohang University of Science and Technology (POSTECH)Pohang37673Korea; ^2^ Bioproduction Research InstituteNational Institute of Advanced Industrial Science and TechnologyTsukubaJapan; ^3^ School of Interdisciplinary Bioscience and BioengineeringPOSTECHPohang37673Korea; ^4^ Division of Integrative Bioscience and BiotechnologyPOSTECHPohang37673Korea; ^5^ Department of New Biology and Center for Plant Aging ResearchDGISTDaegu42988Korea; ^6^ Division of Strategic Research and DevelopmentGraduate School of Science and EngineeringSaitama UniversitySaitamaJapan; ^7^ Department of Plant and Microbial BiologyUniversity ZurichZollikerstrasse 107CH‐8008ZürichSwitzerland

**Keywords:** cadmium (Cd), copper (Cu), GLABRA1 ENHANCER BINDING PROTEIN (GeBP) transcription factor, oxidative stress, reactive oxygen species (ROS), root avoidance, split media assay, zinc (Zn)

## Abstract

- Plants reorganize their root architecture to avoid growth into unfavorable regions of the rhizosphere. In a screen based on chimeric repressor gene‐silencing technology, we identified the *Arabidopsis thaliana* GeBP‐LIKE 4 (GPL4) transcription factor as an inhibitor of root growth that is induced rapidly in root tips in response to cadmium (Cd).
- We tested the hypothesis that GPL4 functions in the root avoidance of Cd by analyzing root proliferation in split medium, in which only half of the medium contained toxic concentrations of Cd.
- The wild‐type (WT) plants exhibited root avoidance by inhibiting root growth in the Cd side but increasing root biomass in the control side. By contrast, GPL4‐suppression lines exhibited nearly comparable root growth in the Cd and control sides and accumulated more Cd in the shoots than did the WT. GPL4 suppression also altered the root avoidance of toxic concentrations of other essential metals, modulated the expression of many genes related to oxidative stress, and consistently decreased reactive oxygen species concentrations.
- We suggest that GPL4 inhibits the growth of roots exposed to toxic metals by modulating reactive oxygen species concentrations, thereby allowing roots to colonize noncontaminated regions of the rhizosphere.

## Introduction

Plants are continuously subjected to abiotic stresses. When exposed to sublethal levels of abiotic stresses, plants exhibit an array of morphological, physiological and biochemical responses that allow them to tolerate, acclimate to or avoid these stresses. Plant growth and worldwide crop productivity depend on plants having successful responses to abiotic stresses. The root system of plants is particularly plastic during development, and this allows plants to adapt to their highly heterogeneous and ever‐changing environment. The root system responds readily to environmental stimuli, changing not only the growth of individual roots, as observed in tropic responses, but also altering the resource allocation between different roots, resulting in whole root architecture changes that optimize water and nutrient absorption from the soil.

The changes in root architecture occur when plants adjust the growth of their primary and secondary roots to adapt to the environment (e.g. patchy nutrient distribution in soil). This involves the development of new lateral roots and the inhibition/promotion of primary root growth. For example, localized nitrate supplies induce the proliferation of lateral roots within the nitrate‐rich zone in many species of plants (Hackett, 1972; Drew, 1975; Scheible *et al*., 1997; Zhang & Forde, 1999). Phosphorus deficiency enhances root hair and lateral root formation, and decreases root elongation in many plant species (Drew, 1975; Péret *et al*., 2014). Likewise, sulfur deficiency induces the formation of lateral roots in *Arabidopsis thaliana* plants (Remans *et al*., 2006; Gruber *et al*., 2013). Such changes in growth enable the root to explore increased soil volumes in search of nutrient‐rich patches. Adaptive changes in root architecture do not always require root growth, but can be achieved by modification of the existing root structure. For example some wetland plant species tend to develop air passage cells in existing roots in addition to form adventitious roots to survive oxygen deficiency caused by flooding (Visser *et al*., 1996; Gibberd *et al*., 2001). Restructuring of the whole root architecture is a biologically important phenomenon for plant survival and yield, but has not received sufficient attention and the detailed mechanisms and molecular factors involved in this process have not been revealed.

Cadmium (Cd), a toxic heavy metal pollutant that has adverse effects on both plants and animals, is distributed heterogeneously in soil, like nutrients and essential metals. The local concentration of Cd absorbable to plant roots varies widely in the soil, depending on the physical and biochemical properties of the soil, such as pH, humidity, the presence of other nutrients (e.g. Zn and Pi), and decomposing plant and animal matter, and the composition of the microbial community in the rhizosphere (Boekhold *et al*., 1991; Wang *et al*., 2006; Yi *et al*., 2007; Sharma & Raju, 2013). Plants can withstand heavy metals either by tolerance and/or avoidance mechanisms (Millaleo *et al*., 2010). Heavy metal tolerance can be achieved by detoxification mechanisms (reviewed in Clemens, 2006; Kramer, 2010), such as extrusion, chelation and sequestration. Plants can reduce internal Cd concentrations by exporting it out of the plant or sequestering it in the vacuole, and several transporters have been implicated in these processes (Kim *et al*., 2007; Baxter *et al*., 2003; Buer *et al*., 2010; DalCorso *et al*., 2010; Rascio & Navari‐Izzo, 2011; Park *et al*., 2012).

Furthermore, plants can prevent heavy metals from entering their cellular system (Millaleo *et al*., 2010), either by adjusting the rhizosphere environment by secreting organic acids that chelate heavy metals (Yang *et al*., 2000; Dong *et al*., 2007) or by changing the root architecture (Potters *et al*., 2007; Remans *et al*., 2012; Vitti *et al*., 2014; Mathieu *et al*., 2015). Several studies reported that plants inhibit primary root growth and, instead, enhance lateral root growth in response to Cd (Potters *et al*., 2007; Remans *et al*., 2012; Vitti *et al*., 2014; Mathieu *et al*., 2015), suggesting that plant roots respond to Cd by changing their entire root architecture (Whiting *et al*., 2000; Potters *et al*., 2007; Liu *et al*., 2010; Remans *et al*., 2012). However, even though soil is highly heterogeneous, with organic and inorganic constituents exhibiting a patchy distribution (Hodge, 2004, 2006, 2009), most studies of the plant's response to Cd contamination have been performed under homogeneous Cd supplementation, and therefore, provide only limited insight into root avoidance of Cd toxicity.

Many important factors involved in sensing and foraging of nutrients in a heterogeneous environment have been identified in roots using a split media system that mimics soil heterogeneity (Drew, 1975; Robinson, 1994; Gansel *et al*., 2001; Remans *et al*., 2006; Li *et al*., 2014; Feller *et al*., 2015). The dual affinity nitrate transporter NRT1.1, which both senses nitrate availability (Remans *et al*., 2006) and transports it (Gansel *et al*., 2001), was identified using this system.

In this study, we used the split media method to show that a novel GLABRA1 ENHANCER BINDING PROTEIN (GeBP) transcription factor (TF; GPL4) in *A. thaliana* inhibits root growth in the region of the rhizosphere contaminated with Cd, and thereby promotes the colonization of roots in noncontaminated regions of the rhizosphere. We also show that failure of root inhibition in response to Cd in plants with suppressed GPL4 results in increased Cd accumulation in the aerial parts. Thus, we reveal a novel TF that is required for the root avoidance response to metal stresses such as Cd.

## Materials and Methods

### Analyses of metals toxicity on plant growth

*Arabidopsis thaliana* (L.) Heynh. ecotype Col‐0 wild‐type (WT) and transgenic plants were grown on half‐strength Murashige‐Skoog (0.5×MS) agar plates with 1% sucrose in the absence or presence of the indicated treatment of metals or chemicals, in a controlled environment with a 16 h 22°C : 8 h 18°C, light : dark cycle for the indicated time periods.

In order to examine the effect of Cd toxicity on plant growth on agar plates, seeds were sown and germinated on 0.5×MS agar plates supplemented with 70 μM CdCl_2_ and grown for indicated periods (five plants per genotype and two to three genotypes per plate). The sensitivity of plants to Cd stress was assessed by measuring fresh weights and root lengths of the whole seedlings. In soil experiment, 3‐wk‐old plants were subjected to 1 mM CdCl_2_ treatment for 4 wk by irrigating the plants every fifth day. Photographs were taken 1 wk after the last irrigation. The sensitivity of plants to Cd stress was assessed by measuring fresh weights and chlorophyll. Briefly, leaf samples were extracted in 95% ethanol at 80°C for 10 min. Chlorophyll contents of the extracts were calculated from absorbance values at 647 and 664 nm, using the following equation: Chlorophyll = (7.93 × A_664_ + 19.93 × A_647_)/fresh weight. The concentrations of 70 μM CdCl_2_ and 1 mM CdCl_2_ were chosen based on the concentration‐dependent toxicity tests (Supporting Information Fig. S1).

The effects of excess copper (Cu) and zinc (Zn) and deficient Zn on plant growth on agar plates were tested as described earlier except that 0.5×MS agar medium was supplemented with 65 μM CuCl_2_, 0.5 mM ZnCl_2_ or, 5 μM TPEN (N,N,N′,N′‐Tetrakis‐(2‐pyridylmethyl) ethylenediamine), a Zn chelator.

### Identification of transcription factors involved in the Cd response using *A. thaliana* chimeric repressor gene‐silencing technology (CRES‐T) lines

Chimeric repressor gene silencing technology (CRES‐T) is a novel gene silencing system in which a dominant negative repressor of a TF suppresses the target genes of endogenous TF and its functionally redundant TFs (Hiratsu *et al*., 2003). Pooled seeds of *c*. 1600 *A. thaliana* CRES‐T lines were sown on 0.5×MS medium supplemented with 70 μM CdCl_2_, and Cd‐tolerant plants were selected for further analysis. To identify the transcription factor affected in Cd‐tolerant CRES‐T plants, genomic DNA was extracted, and transgenes were amplified using a forward primer that binds to the 35S promoter and a reverse primer that binds to the repressor domain, SRDX (Table S1). The PCR product was purified using a LaboPass TM GEL and PCR Clean‐up Kit, and sequenced.

### Generation of GPL4 transgenic *A. thaliana* plants

RNAi lines in which GPL4 is silenced by RNA intereference were generated by transforming *A. thaliana* plants (Col‐0 ecotype) with a pHellsgate8 binary vector containing a gene‐specific sequence (Wesley *et al*., 2001). Lines overexpressing GPL4 (OX) were generated using a pCambia1302 vector containing an sGFP fusion of the *GPL4* coding sequence (CDS) driven by the 35S promoter (*35S*_*pro*_ *:GPL4:sGFP*). To analyze the tissue‐specific expression of *GPL4*,* A. thaliana* plants were transformed by a pBI121 binary vector containing the *GPL4* CDS in a translational fusion with GUS or sGFP driven by the native promoter (*GPL4*_*pro*_ *:GPL4:GUS* and *GPL4*_*pro*_ *:GPL4:GFP*, respectively).

### Analysis of Cd‐mediated *GPL4* induction

Liquid 0.5×MS medium containing 70 μM CdCl_2_ was poured over the roots of 7‐d‐old WT or *GPL4*_*pro*_ *:GPL4:GUS* and *GPL4*_*pro*_ *:GPL4:sGFP* plants grown on 0.5×MS agar medium. After the indicated periods of incubation, GPL4 expression levels were assessed by quantitative reverse transcription polymerase chain reaction (qRT‐PCR) analysis (with *Tubulin8* as an internal control) or by monitoring GUS expression or GFP fluorescence. Primers used for qRT‐PCR and transgenic generation are listed in Table S1. The expression level of the reference gene *Tubulin 8* did not significantly change upon Cd treatment.

### ROS measurement

ROS were detected using the dye H_2_DCFDA (2′,7′‐dichlorodihydrofluorescein diacetate) as previously described (Lee *et al*., 1999). One‐ to two‐week‐old seedlings were incubated for 60 min at 4°C in 0.5×MS medium containing 10 μM DCF‐DA and then in liquid 0.5×MS supplemented or not with 70 μM CdCl_2_ for 60 min at 22°C. After washing with 0.1 mM KCl and 0.1 mM CaCl_2_ (pH 6.0) solution, dichlorodihydrofluorescein (DCF) fluorescence was observed by fluorescence microscopy and quantified (ImageJ). To quantify DCF fluorescence at the whole seedling level, seedlings treated as described earlier were ground and extracted using 1 ml of 10 mM Tris‐HCl (pH 7.2) and 1% Triton X‐100. From the supernatant obtained by centrifugation at 4°C for 20 min, protein content and DCF fluorescence intensity were measured using Bradford's assay and a Tecan spectrophotometer (Infinite M200PRO), respectively.

### Transcriptome analysis of GPL4 CRES‐T plants

Expression analysis of the WT and CRES‐T lines with or without Cd treatment was performed using Agilent Arabidopsis (V4) Gene Expression Microarrays (Agilent, Santa Clara, CA, USA) with two independent replicates. RNAs were extracted from 2‐wk‐old WT and CRES‐T seedlings treated or not with 70 μM CdCl_2_ for 2 h using an RNA Prep Kit (Qiagen, Manchester, UK). Signals were analyzed using Agilent Feature Extraction software (v.7.5.1), and intensities were normalized using the quantile normalization method (Bolstad *et al*., 2003) after log_2_ scale conversion. To identify present probes, a Gaussian mixture model (one for present probes and the other for absent probes) was fitted to the distribution of the quantile normalized log_2_ intensities. Probes with intensities larger than a cut‐off in which two Gaussian distributions met were determined to be present.

Differentially expressed genes (DEGs) were identified using the integrative statistical method described previously (Chae *et al*., 2013). Briefly, *t*‐statistics were computed from the two‐tailed *t*‐test for the two sample groups (CRES‐T and WT) with the assumption of unequal variance, and median differences between the two sample groups were also computed; empirical null distributions for the *t*‐statistics and the median difference were derived from random permutations of the whole samples; *P*‐values for *t*‐statistic values and median differences for individual genes were computed based on the corresponding empirical null distributions; two *P*‐values for *t*‐statistic values and median differences were combined using the Liptak‐Stouffer Z method (Hwang *et al*., 2005); and DEGs were finally selected as the genes with a combined *P*‐value of < 0.05 and fold change of ≥ 1.5.

For the enrichment analysis of gene ontology biological processes (GOBPs), the DEGs were first grouped into eight clusters based on whether they were up‐ or downregulated in CRES‐T, compared with the WT, in the presence and absence of Cd treatment. The GOBP enrichment analysis was then performed for the DEGs in individual clusters using DAVID software (Huang *et al*., 2009). The GOBPs significantly represented by the genes in individual clusters were selected based on having an enrichment *P*‐value of < 0.05 computed from DAVID.

Transcript level changes of selected DEGs after 2 h of Cd treatment were confirmed by qRT‐PCR analysis using gene‐specific primers (Table S1). *Tubulin8* was used as an internal control. The transcript level of *Tubulin8* was not affected by Cd treatment.

### Split media assay

In the vertically split 0.5×MS agar media assay, primary roots of 4‐d‐old seedlings were removed and the seedlings were grown for a further 8 d to develop two‐first‐order lateral roots. Twelve‐day‐old seedlings with two lateral roots of similar length were transferred to split plates containing 0.5×MS media, with one lateral root per side. In the split root plate, one side of the 0.5×MS agar media was supplemented with 10–30 μM CdCl_2_, 35 μM CuCl_2_, 0.5 mM ZnCl_2_, 5 μM TPEN, 5 nM PQ, or 60 or 80 mM NaCl (final concentration), whereas the other side was supplemented with an equal volume of water. Growth parameters were analyzed a week after transfer. In the horizontally split 0.5×MS agar media assay, the bottom half was supplemented with 10–70 μM CdCl_2_ and the top half supplemented with an equal volume of water. We used Cd concentrations as low as 10 μM, based on our concentration dependency test and a previous report (Remans *et al*., 2012).

Rhizoboxes for the split soil assay were prepared according to Whiting *et al*. (2000), with modifications. Square culture plates were partitioned into two chambers using plastic barriers and filled with soil moistened with water or Cd‐containing water (final concentrations of 1, 3 and 6 mM). Twelve‐day‐old seedlings with two lateral roots, generated as described earlier, were transferred to each box, with one root on each side (just above the soil surface), and the shoot was exposed through a slit in the lid. The individual boxes were sealed with 3M surgical tape, covered with aluminum foil, and incubated in a controlled environment. After 3 wk, photographs were taken and shoot weight and Cd content were measured.

### Measurement of heavy metal contents

In order to measure the contents of Cd, Cu and Zn in *A. thaliana* plants grown under Cd stress, plants were grown on 0.5×MS agar medium supplemented with 10 μM of Cd for 2 wk and washed thoroughly twice with 1 mm CaCl_2_ solution and then once with ice‐cold water. Samples were then completely digested with 65% HNO_3_ at 100°C and diluted with distilled water. Cd, Cu and Zn contents were measured by Inductively Coupled Plasma Spectrometry (ICP‐MS; Perkin Elmer, San Jose, CA, USA).

## Results

### A GeBP transcription factor is critical for Cd‐induced growth inhibition

**Figure 1:**
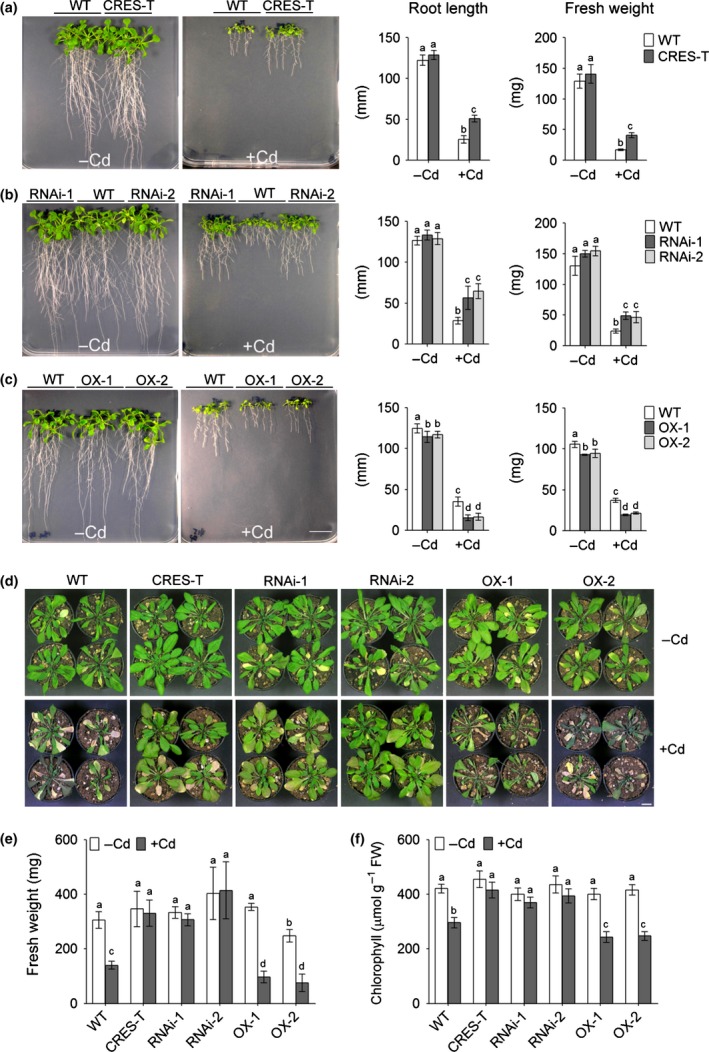
Genetically modified *Arabidopsis thaliana* plants with suppressed GPL4 function or altered *GPL4* expression exhibited altered sensitivity to cadmium (Cd) stress. (a–c) Enhanced Cd tolerance of plants in which GPL4 was suppressed by chimeric repressor gene silencing technology (CRES‐T, a) or RNA interference (RNAi, b) and (c) enhanced Cd sensitivity of overexpressing GPL4 (OX) plants compared with the wild‐type (WT). Plants were grown on 0.5×MS agar plates without or with supplementation of 70 μM CdCl_2_ (−Cd and +Cd, respectively) for 2 wk. Bar, 1 cm. Data of three independent experiments were combined and mean values (± SE) of the longest root length and total fresh weight per plate are presented. Each individual experiment was performed with at least four plates per genotype and treatment. (d–f) Altered Cd sensitivity of GPL4 transgenic (CRES‐T, RNAi and OX) lines grown in soil. Plants were watered without or with 1 mM CdCl_2_ (−Cd and +Cd, respectively) every fifth day until photographs were taken (d). Data of two independent experiments were combined and mean values (± SE) of (e) fresh weight and (f) chlorophyll content of the whole rosette are presented. Each experiment was performed with ≥ 4 plants per treatment and genotype. Bar, 1 cm. Different letters indicate that the means are significantly different between genotypes and treatments by Tukey's Honest Significant Difference test at *P *≤* *0.05 (a–c, e, f).

In order to identify the TF(s) that play an important role in the plant's response to Cd, we screened *c*. 1600 *A. thaliana* transgenic lines that expressed chimeric repressors (CRES‐T) for lines that grew better than the WT on 0.5×MS medium supplemented with 70 μM CdCl_2_ (Fig. S1). We identified a novel TF belonging to the GeBP family (At1g44810, Fig. 1a) and confirmed the Cd tolerance with independently generated CRES‐T lines. We named this TF GeBP‐LIKE 4 (GPL4) because it is the fourth GeBP‐like TF to be identified in *A. thaliana* (Curaba *et al*., 2003; Chevalier *et al*., 2008; Perazza *et al*., 2011).

In order to confirm that Cd tolerance was due to GPL4 suppression, we generated GPL4 knockdown lines using RNA interference (RNAi) technology. We tried to isolate homozygous knockouts from the available T‐DNA insertion lines (SALK_020363 and SALK_038910), but failed. In the RNAi lines, the expression of *GPL4* was specifically downregulated (Fig. S2). Similar to the CRES‐T lines, the RNAi lines also exhibited Cd tolerance (Fig. 1a,b); in the presence of Cd, CRES‐T and RNAi produced longer roots and larger biomass than the WT. By contrast, lines that overexpressed GPL4 (OX, *35S*_*pro*_ *:GPL4:sGFP*) exhibited shorter roots and less biomass than the WT (Cd hypersensitivity; Fig. 1c).

Soil‐grown GPL4 transgenic plants exhibited similarly altered Cd toxicity (Fig. 1d–f). CRES‐T and RNAi plants exhibited tolerance to Cd, whereas OX plants exhibited hypersensitivity to Cd (Fig. 1d–f). Cd treatment did not affect the shoot biomass (Fig. 1e) or chlorophyll content (Fig. 1f) of CRES‐T and RNAi lines. By contrast, Cd treatment greatly reduced the production of biomass and chlorophyll in the WT and OX lines (Fig. 1e,f), and the effect was slightly more pronounced in the OX lines than in the WT, as can be seen in chlorophyll contents (Fig. 1f). Taken together, our results indicate that GPL4 plays an important role in Cd‐induced growth inhibition.

The plants shown in Fig. 1(d) were photographed after a week of withholding water supply. Thus, plants were wilted, but to different extents depending on the genotypes. Because severe wilting was apparent only in WT and OX plants treated with Cd, wilting seems to be a trait controlled, at least in part, by the GPL4. Water status in plants is known to be highly sensitive to Cd (Barcelö *et al*., 1986; Perfus‐Barbeoch *et al*., 2002). However, in the following analyses, we focused mainly on root length and fresh weight phenotypes, which are traits that can be readily assayed in many replicates and are thus amenable to rigorous statistical analysis.

### GPL4 is induced in the root tips in response to Cd

**Figure 2:**
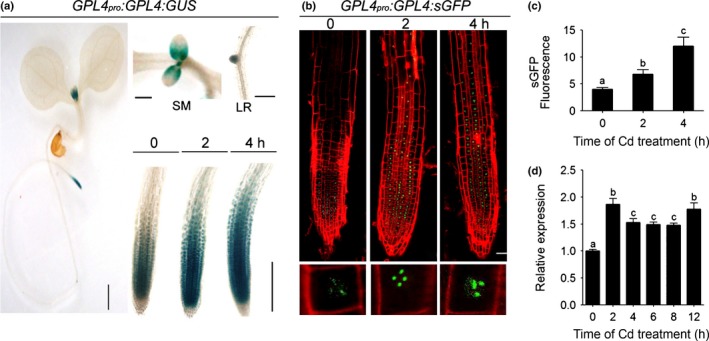
GPL4 is induced by cadmium (Cd) treatment in *Arabidopsis thaliana* root tips. (a) Tissue‐specific expression of GPL4 in 6‐d‐old *GPL4*_*pro*_ *:GPL4:GUS* seedlings and induction by Cd. Whole seedling (left panel; bar,1 mm); shoot meristem (SM, upper middle panel; bar, 500 μm); emerged lateral root (LR, upper right panel; bar,100 μm); and induction in the root tip observed at three time points (0, 2 and 4 h) after 70 μM CdCl_2_ treatment (lower right panel; bar, 100 μm). (b, c) Cd‐mediated induction of GPL4 in *GPL4*_*pro*_ *:GPL4:sGFP* seedlings, observed at three time points (0, 2 and 4 h) after 70 μM CdCl_2_ treatment (b, upper panel). Bar, 20 μm. An enlarged view of an individual cell with representative GPL4:sGFP expression level at indicated time points (b, lower panel); GPL4:sGFP was observed as prominent speckles in the nucleus. Relative fluorescence intensity (in arbitrary units) of sGFP in the root tips (c). Mean values (± SE) of three independent observations with ≥ 10 seedlings per time per experiment are shown. (d) Rapid increase in *GPL4* transcript level upon treatment with 70 μM CdCl_2_. Transcript levels were normalized by *beta tubulin 8* as an internal control. Mean values (± SE) of five independent experiments with three replicates per time point per experiment are shown. Different letters indicate that the means are significantly different between each time point by Tukey's Honest Significant Difference test (*P *≤* *0.01).

We then characterized the tissue‐specific expression of GPL4 using *GPL4*_*pro*_ *:GPL4:GUS* lines. GPL4 expression was detected in the tips of the main and lateral roots and apical meristem of shoots (Figs 2a, S3a). GPL4 expression was also observed in the young floral buds, siliques, developing embryos and senescing leaves (Fig. S3a). We confirmed the expression of GPL4 in the root tips using *GPL4*_*pro*_ *:GPL4:sGFP* (Fig. 2b). GPL4:sGFP was localized as punctate speckles within the nucleus in the root tip (Fig. 2b), which was confirmed with the DAPI nuclear stain.

TFs involved in a stress response are often induced by that stress (Wang *et al*., 2016). Indeed, GPL4 was significantly induced within 2 h of Cd treatment at both transcript and protein levels (Fig. 2a–d). The root tip expression and induction by Cd suggest that GPL4 is involved in the early response to Cd stress in the rhizosphere.

### GPL4 regulates root biomass reallocation to avoid Cd toxicity

The question then arose as to why plants need a gene that renders them more sensitive to stress. We hypothesized that GPL4 plays a role in the Cd avoidance response (i.e. reorganizing root architecture to avoid Cd toxicity). To test this hypothesis, we investigated root proliferation of the WT and GPL4 transgenic plants in split media assays, which simulate the heterogeneity of Cd contamination in fields.

**Figure 3:**
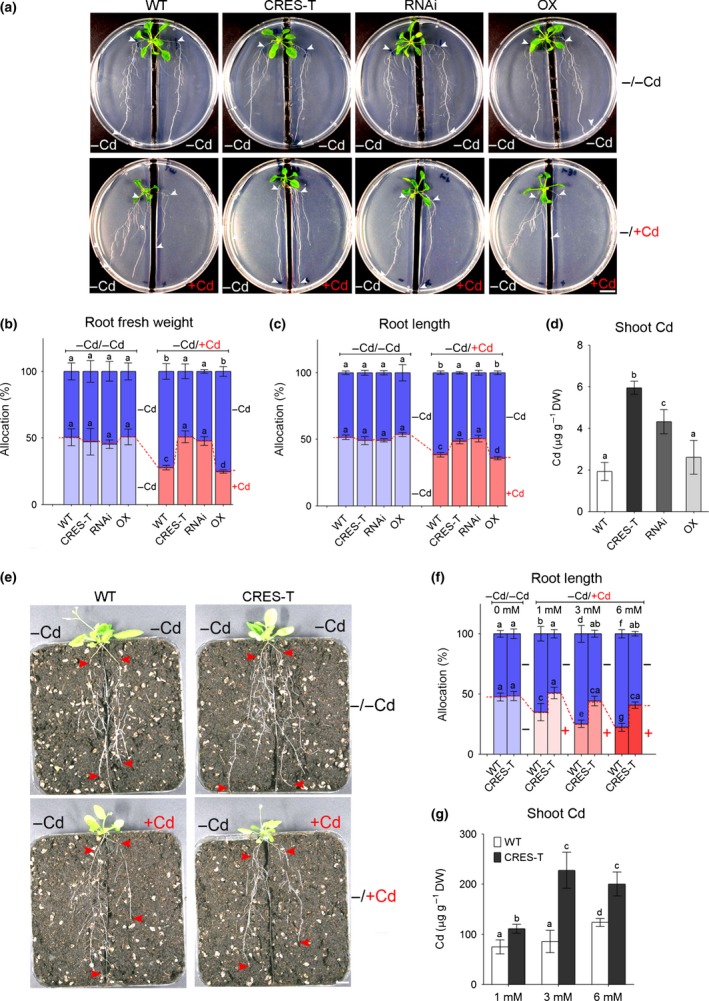
GPL4 limits root growth on cadmium (Cd)‐containing medium and increases root growth on normal medium in *Arabidopsis thaliana*. (a–d) Growth of wild‐type (WT) plants, GPL4 suppressed plants by chimeric repressor gene silencing technology (CRES‐T) or RNA interference (RNAi), and GPL4 overexpression (OX) plants on vertically split media supplemented with water (−Cd) and 10 μM CdCl_2_ (+Cd). Representative images of homogeneous (−/−Cd; upper panel) and heterogeneous (−/+Cd; lower panel) Cd supplementation (a). Arrowheads indicate net growth of the main roots after transfer. Bar, 1 cm. Mean percentage allocation (± SE) of (b) root fresh weight and (c) the longest root length on the two sides of the split media from three independent experiments. The percentage values in the left (−Cd) are in the upper segments of the bars (blue) and in the right (−Cd for ‘−/−Cd’; light blue and +Cd for ‘−/+Cd’; pink) are in the lower segments. Cd contents in the shoots of plants grown in heterogeneous (−/+Cd) split media (d). Each individual experiment was performed with ≥ 6 seedlings per genotype per treatment. (e, f) Growth of WT and CRES‐T plants in rhizoboxes. Representative images (e) of homogeneous (−/−Cd) control soil in both chambers (top), and heterogeneous (−/+Cd) soil supplemented with 1 mM Cd in the right side (bottom). Arrowheads indicate net growth of the main roots after transfer to rhizoboxes. Bar, 1 cm. Mean percentage allocation (± SE) of the longest root length between the two chambers in rhizoboxes analyzed at three different concentrations (1 mM, 3 mM, and 6 mM) of CdCl_2_ are presented (f). The percentage values in the left (−Cd) are in the upper segments of the bars (blue) and those in the right (−Cd for ‘−/−Cd’; light blue and +Cd for ‘−/+Cd’; pink, increase in color intensity denotes an increase in Cd concentration) are in the lower segments. Cd contents in shoots of plants grown in heterogeneous soil containing 1 mM, 3 mM, or 6 mM CdCl_2_ in right rhizobox chamber and no Cd in the left (g). The assay was performed three times with ≥ 6 seedlings per genotype per treatment and per assay. Different letters indicate that the means are significantly different between genotypes and treatments by Tukey's Honest Significant Difference test at *P *≤* *0.05 (b–d, f, g).

First, we examined root growth in agar media with two vertically split halves, one of which was filled with 0.5×MS agar medium supplemented with 10–30 μM CdCl_2_, and the other was filled with regular 0.5×MS agar medium (Fig. 3a). The WT and GPL4 transgenic (CRES‐T, RNAi, and OX) plants with two first‐order lateral roots of similar size were transferred to the vertically separated media and grown for further 8 d. In control plates with no Cd in either side (−/−Cd), root biomass was equal on both sides for all four genotypes (Figs 3a–c, S4; Table S2). However, in the plates containing two different media (−/+Cd), the WT and OX roots exhibited severe growth retardation in the Cd side, but strong growth in the Cd‐free control side (Figs 3a–c, S4; Table S2). By contrast, in CRES‐T and RNAi plants, retardation of root growth in the Cd side was either negligible (Fig. 3a–c; Table S2; 10 μM of Cd) or suppressed dramatically (Fig. S4; Table S2; 30 μM of Cd), and root growth in the Cd‐free side was not promoted (Figs 3a–c, S4; Table S2). We obtained similar results in a split medium assay using soil‐grown plants in rhizoboxes, which were filled with Cd‐contaminated soil on one side of a partition and normal soil on the other (Fig. 3e,f; Table S3).

**Figure 4:**
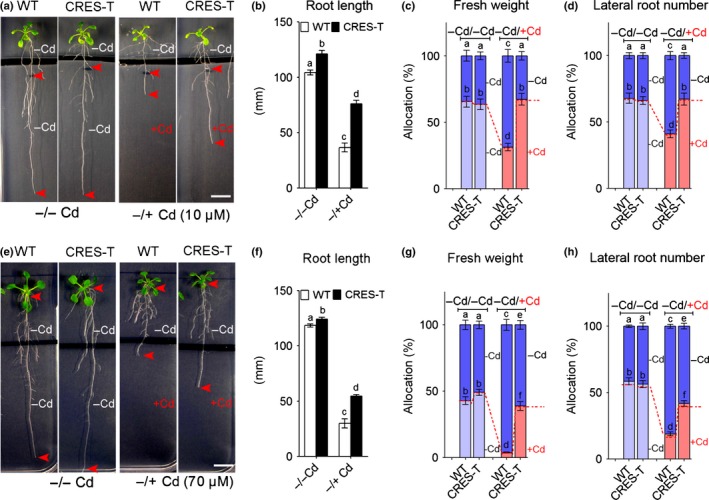
GPL4 reallocates root growth from the cadmium (Cd)‐containing medium towards normal medium in *Arabidopsis thaliana*. (a–d) Growth of wild‐type (WT) plants and GPL4 suppressed plants by chimeric repressor gene silencing technology (CRES‐T) in horizontally split medium containing 10 μM CdCl_2_ in the bottom half. (a) Representative images of homogeneous (−/−Cd, left panels) and heterogeneous (−/+Cd; right panels) medium. Intact 5‐d‐old seedlings were transferred to the split media, and arrowheads indicate net main root growth after transfer. Lateral roots of the WT in heterogeneous (−/+Cd) medium were artificially straightened. Mean values (± SE) of three independent experiments are given: (b) net primary root growth, (c) percentage allocations of the root fresh weight and (d) lateral root numbers between the top and bottom media. (e–h) Growth of WT and CRES‐T plants in horizontally split medium containing 70 μM CdCl_2_ in the bottom half. Representative images of homogeneous (−/−Cd, left panels) and heterogeneous (−/+Cd; right panels) medium (e). Seeds were directly sown on split media. Mean values (± SE) of three independent experiments are given; (f) primary root length, (g) percentage allocations of the root fresh weight and (h) lateral root numbers between the top and bottom media. The percentage values in the top (−Cd) are represented in the upper segments (blue) of the bars and those in the bottom (−Cd for ‘−/−Cd’; light blue and +Cd for ‘−/+Cd’; pink) are in the lower segments (c, d, g, h). Each individual experiment was performed with ≥ 8 seedlings per genotype and treatment. Different letters indicate that the means are significantly different between genotypes and treatments by Tukey's Honest Significant Difference test (*P *≤* *0.05). Bars, 1 cm.

We confirmed the results by placing intact seedlings or sowing seeds directly on horizontally split agar medium, in which the bottom half was supplemented with 10 and 70 μM CdCl_2_ and the top half contained control medium (Fig. 4; Table S4). The WT plants inhibited primary root growth in the Cd‐containing media, and instead, proliferated lateral roots in the top half, which lacked Cd. By contrast, CRES‐T plants failed to exhibit such changes in root proliferation (Fig. 4a–d). Similar but more pronounced differences between CRES‐T and the WT were observed at higher Cd concentration (70 μM CdCl_2_, Fig. 4e–h).

These results indicate that GPL4 is necessary for root avoidance response, that is, inhibition of root growth in the Cd‐contaminated medium and colonization in the noncontaminated medium.

### GPL4‐mediated root avoidance is necessary to reduce Cd accumulation in the shoots

Many toxic metals enter roots through essential metal transporters (Clemens, 2006). Because the survival of the plant depends on the root's ability to absorb essential metals, absorption of toxic metals in the environment cannot be avoided completely. We suspected that the large root surface area observed for CRES‐T and RNAi plants grown in media contaminated with Cd might result in increased Cd uptake and accumulation in the plants. Indeed, the Cd contents of CRES‐T and RNAi shoots was two‐ to three‐fold higher than those of the WT and OX shoots, both for plants grown on agar medium (Fig. 3d) and in soil (Fig. 3g), when only half of the growth medium was contaminated with Cd. The shoot Cd content increased with increasing concentrations of Cd in the soil (Fig. 3g). These results suggest that the WT plants limit Cd accumulation in their shoots by reducing the proliferation of roots in Cd‐contaminated regions of the growth medium and that this depends on GPL4.

### GPL4 transcriptionally regulates the expression of oxidative stress‐related genes

In order to get insight on the mechanism of GPL4‐mediated root avoidance, first of all, we determined the GPL4 activity as a transcription factor using a transient expression assay in *A. thaliana* rosette leaves (Fig. S3b; Methods S1). Fusion of GPL4 to the GAL4 DNA‐binding domain (GAL4DB‐GPL4) significantly enhanced the expression of the reporter luciferase compared with the GAL4DB control (Fig. S3b, *P *<* *0.01). Similar transcriptional activation activity of GPL4 was observed by another reporter system in yeast (Fig. S3c; Methods S2).

**Figure 5:**
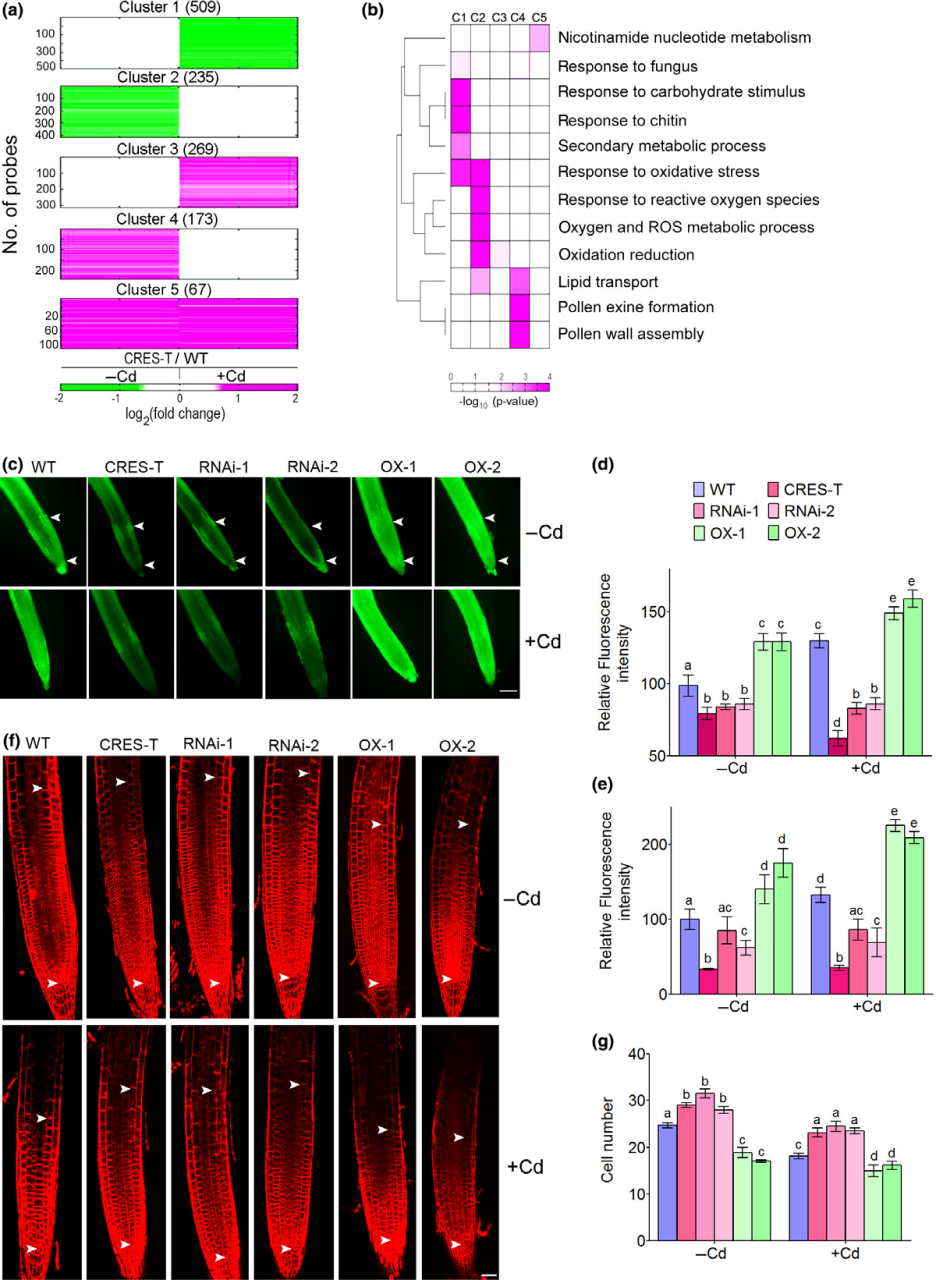
GPL4 regulates the expression of oxidative stress‐related genes, reactive oxygen species (ROS) concentration, and the cell number at the root apical meristem (RAM) in *Arabidopsis thaliana*. (a) Differentially expressed genes (DEGs) between wild‐type (WT) plants and GPL4 suppressed plants by chimeric repressor gene silencing technology (CRES‐T) in the absence (−Cd) and presence (+Cd) of 70 μM CdCl_2_ were grouped into five major clusters (Clusters 1–5). Green and magenta indicate down‐ and upregulation in CRES‐T compared with the WT, respectively. The color bar represents the gradient of log_2_‐fold changes between CRES‐T and the WT. Numbers in parentheses indicate the number of DEGs. (b) Gene ontology biological processes represented by DEGs in Clusters 1–5 (C1–C5). The color represents the significance (*P *<* *0.05) of each process being enriched by the genes in individual clusters. The color bar shows the gradient of −log_10_(*P*‐value), where the *P*‐value is the enrichment significance computed by DAVID. The dendrogram shows the clustering results of the processes. (c–e) ROS concentration assessments using H_2_ DCF‐DA dye without (−Cd) and with (+Cd) 70 μM CdCl_2_ treatment. Representative images of root tips are shown (c). Arrowheads indicate the local ROS maxima in the −Cd condition. The mean values (± SE) of DCF fluorescence measurement (normalized to WT value in the −Cd condition) in (d) the root tip and (e) whole seedlings were obtained from three independent experiments. Each experiment was performed with 10 seedlings per time point and genotype. (f, g) Size of the root apical meristem in 7‐d‐old WT and GPL4 transgenic seedlings grown on 0.5×MS media without or with Cd supplementation (−Cd and +Cd, respectively). (f) Representative root tip images and (g) mean cortical cell numbers (± SE) in the root apical meristems obtained from three independent observations are shown. Arrowheads indicate the root apical meristems. Each observation was performed with five seedlings per genotype per treatment. Different letters indicate that the means are significantly different between genotypes and treatments by Tukey's Honest Significant Difference test at *P *≤* *0.01 (d, e, g). Bars: (c) 50 μm; (f) 20 μm.

We next identified potential target genes of GPL4 by comparing the genome‐wide gene expression profiles of GPL4 CRES‐T and the WT plants treated or not with 70 μM CdCl_2_ (Fig. 5a). We identified a total of 1289 differentially expressed genes (DEGs) between CRES‐T and the WT plants in the absence or presence of Cd (see the Materials and Methods section for details). We then grouped 1289 DEGs into eight clusters and focused on the five major clusters that contained > 50 DEGs (i.e. Clusters 1–5 in Fig. 5a; Table S5): Clusters 1 and 3 included genes that were suppressed and induced in GPL4 CRES‐T plants vs in the WT in the presence of Cd, respectively; Clusters 2 and 4 included genes that were suppressed and induced in CRES‐T vs in the WT in the absence of Cd, respectively, but that exhibited similar expression in the wild type and CRES‐T in the presence of Cd; and Cluster 5 included genes that were induced in CRES‐T vs in the WT both in the presence and absence of Cd. Strikingly, many genes in Clusters 1 to 3 are involved in the oxidative stress response according to our gene ontology biological processes analysis (Fig. 5b; Table S6). We evaluated the expression levels of a subset of these genes by qRT‐PCR (Fig. S3d,e) and confirmed that their expression levels were significantly up‐ or downregulated in GPL4 CRES‐T by Cd stress (*P *<* *0.01).

Cluster 1 most likely includes direct targets of GPL4 in Cd response. We therefore further investigated 10 genes from Cluster 1 (Table S6; Note S1) to find out if they are under direct control of GPL4. We examined the interaction between GPL4 and promoters of 10 genes using yeast‐one‐hybrid (Y1H) and Luciferase reporter activity assays (Methods S1, S2) and found strong interaction of GPL4 to the promoter of *OXYSTEROL‐BINDING PROTEIN* (*OSBP*)*‐RELATED PROTEIN 4C* (*ORP4C*) and weak interaction to the promoters of an *ETHYLENE RESPONSIVE TF family member* (*ERF61*) and a *2‐OXOGLUTARATE‐IRON‐DEPENDENT* (*2OG‐Fe(II)‐dependent*) *OXYGENASE* family member (Fig. S5a,b). We evaluated the role *ERF61* and *ORP4C* in the Cd stress response using their knockout mutants (*erf61* and *orp4c*, respectively). *The 2og‐fe(II)‐dependent oxygenase* knockout mutant was not available at the time of the study. In the presence of 70 μM CdCl_2_, *orp4c* and *erf61* plants grew better (both root length and fresh weight) than the WT, similar to GPL4 CRES‐T plants (Fig. S5c).

Taken together, these results suggest that GPL4 regulates the expression of genes involved in oxidative stress response to Cd stress either directly or indirectly.

### GPL4 regulates ROS concentration and cell number in the root meristem

We then examined the physiological relevance of the transcriptomic data (Fig. 5a,b). First, ROS concentration was analysed in the GPL4 transgenic lines (CRES‐T, RNAi, OX) in response to Cd exposure. We measured ROS concentrations in the roots, specifically in the tip regions, where *GPL4* is expressed, using H_2_DCFDA dye (Fig. 5c). H_2_DCFDA is deacetylated in the cell and oxidized by ROS, producing highly fluorescent DCF (Kooy *et al*., 1997). Exposure to Cd induced an increase in DCF fluorescence in the root tips and whole seedlings of the WT plants (Fig. 5c–e). However, the Cd‐induced increase of DCF fluorescence was markedly suppressed in CRES‐T and RNAi lines, whereas it was enhanced in OX lines (Fig. 5c–e). Moreover, we observed slight differences in ROS concentrations between genotypes under control (−Cd) conditions (Fig. 5d,e). We also found that the Cd‐induced ROS production after Cd treatment was also significantly reduced in *erf61* and *orp4c* root tips and whole seedlings of *erf61* compared with the WT (Fig. S5d).

Second, we investigated whether GPL4 regulates the growth of the root apical meristem (RAM) because high concentrations of ROS inhibit cell proliferation (Sanz *et al*., 2012), and more specifically, the ROS generated by Cd stress inhibit root growth (Tamas *et al*., 2010). As shown in Fig. 5f, CRES‐T and RNAi lines exhibited longer meristematic zones (more cells), whereas OX lines exhibited shorter meristematic zones (fewer cells) than the WT in both the presence and absence of Cd treatment (Fig. 5f,g; with no significant difference in cell length). Consistent with altered meristem activity, root length was significantly different between 1‐ to 2‐wk‐old WT and GPL4 transgenic seedlings, even in the absence of Cd (Fig. S6). Taken together, these results indicate that GPL4 plays an important role in ROS generation at the root tip. Furthermore, the results suggest that the decreased ROS concentrations in CRES‐T and RNAi diminish the Cd‐induced inhibition of RAM activity, enabling continuous root growth in media contaminated with Cd.

### GPL4 regulates the generation of ROS, which in turn inhibits root growth

In order to further test our hypothesis that ROS generation is a central mechanism of GPL4‐mediated root growth inhibition in Cd stress, we applied an ROS inducer, paraquat (PQ; Bus & Gibson, 1984), to one‐half of a vertically split plate containing 0.5×MS agar medium. We found that the WT root growth was severely inhibited in the PQ side (Fig. S7a–c; Table S7), but was enhanced in the control side. The ROS inducer also significantly inhibited root growth of CRES‐T. However, it was to a lesser extent than in the WT and no enhancement of root growth in the control side (Fig. S7a–c).

We then tested whether quenching ROS can recover OX from the Cd hypersensitivity using an ROS quencher, potassium iodide (KI; Tsukagoshi *et al*., 2010). KI treatment cancelled the hypersensitive response of OX lines to Cd with respect to total fresh weight and root length (Fig. S7d), and reduced ROS concentrations in the OX root tips to the WT level (Fig. S7e). These data indicate that ROS is involved in the GPL4‐mediated root growth inhibition and thereby allowing root colonization in favourable growth conditions.

### GPL4 does not directly modulate Cd transport or glutathione‐mediated tolerance

Our results suggest that GPL4 regulates ROS concentrations during Cd response (Fig. 5). However, we could not exclude the possibility that other important Cd tolerance mechanisms, such as Cd transport and chelation, might be regulated by GPL4. First, to examine whether Cd uptake and translocation was compromised in the GPL4 transgenic lines, we measured the Cd contents from seedlings grown on homogeneous medium containing 10 μM CdCl_2_ for 2 wk (Fig. S8). To avoid any effect caused by large differences in the biomass of the plants, we used 10 μM CdCl_2_, which did not significantly affect plant growth. However, we did not detect any difference in Cd contents of roots, shoots and whole seedlings between the genotypes (Fig. S8a–c). Second, we measured Zn and Cu contents in Cd‐treated WT and GPL4 transgenic lines, because Cd competes with and replaces essential metals producing toxicity. However, neither Zn nor Cu contents differed between the genotypes (Fig. S8d,e). Third, we tested the involvement of glutathione, which plays an important role in Cd chelation and tolerance (Cobbett & Goldsbrough, 2002), using glutathione biosynthesis inhibitor, buthionine sulfoximine (BSO). In the presence of BSO, CRES‐T and OX plants still exhibited Cd tolerance and hypersensitivity, respectively (Fig. S8f–k). Taken together, these results indicate that it is unlikely that GPL4 regulates cellular mechanisms involved in transport or glutathione‐dependent chelation of Cd.

### GPL4 is required for root avoidance in response to stress by essential metals

**Figure 6:**
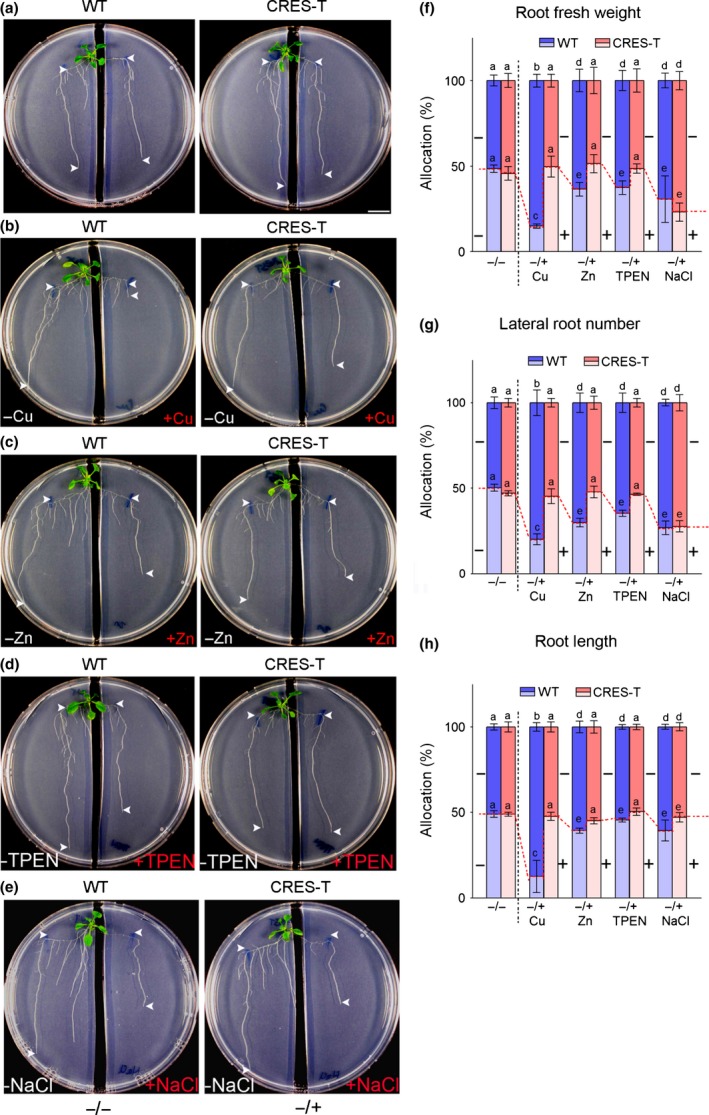
GPL4 is necessary for the root avoidance response to essential metal stresses in *Arabidopsis thaliana*. (a–e) Growth of wild‐type (WT) plants and GPL4 suppressed plants by chimeric repressor gene silencing technology (CRES‐T) on vertically split media supplemented with (a) water in both sides, or with water in the left side and (b) 65 μM of CuCl_2_ (+Cu), (c) 0.5 mM of ZnCl_2_ (+Zn), (d) 5 μM of TPEN or (e) 60 mM NaCl in the right side. Representative images from three independent experiments are shown. Arrowheads indicate net growth of the main roots after transfer. Bar, 1 cm. (f–h) Mean percentage allocation (± SE) of (f) root fresh weight, (g) lateral root number and (h) longest root length between the two sides of the split media for seedlings tested in (a–e). The percentage values in the left (−) are in the upper segments (blue for WT; pink for CRES‐T) of the bars and those in the right (light blue for WT; light pink for CRES‐T) are in the lower segments. Combined results of three independent experiments are given. Each experiment was performed with ≥ 6 seedlings per genotype per treatment. Different letters indicate that the means are significantly different between genotypes and treatments by Tukey's Honest Significant Difference test (*P *≤* *0.05).

Our data indicate that GPL4 is involved in the ROS‐mediated root growth inhibition and GPL4 suppression (CRES‐T and RNAi) protects roots from Cd‐induced oxidative stress. An increase in ROS concentrations is a common toxicity response to various metal stresses, such as Cu or Zn excesses and Zn deficiencies (Sharma *et al*., 2004; D'Souza & Devaraj, 2012; Saha *et al*., 2012). Therefore, we were curious whether GPL4 is also involved in toxicity responses to other metals. First, we examined growth in homogeneous growth medium supplemented with excess Cu, excess Zn and Zn chelator (Fig. S9). CRES‐T and RNAi lines exhibited tolerance and OX lines exhibited hypersensitivity to excess Cu, excess Zn, and Zn deficiency (Fig. S9a–e). Moreover, *GPL4* expression was increased in response to excess Cu and Zn (Fig. S9f). Second, we assessed the root avoidance response of CRES‐T plants grown in the presence of excess or deficient concentrations of essential metals in a split media system (Fig. 6). The WT roots clearly exhibited the avoidance response to excess Cu, excess Zn, Zn deficiency, and salt stress and colonization in the control chambers (Fig. 6a–h; Table S8). However, CRES‐T plants failed in the avoidance response to metal stresses but not to salt stress (Fig. 6a–h, Table S8). Taken together, these results suggest that GPL4 mediates root avoidance following exposure to diverse metal stresses, but not to salt stress.

## Discussion

**Figure 7:**
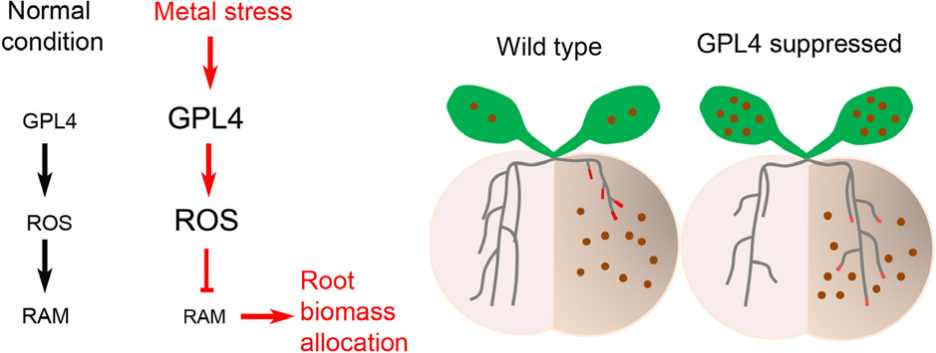
Hypothetical model of GPL4 function in *Arabidopsis thaliana*. GPL4 regulates root avoidance of heavy metal‐contaminated soil patches by regulating reactive oxygen species (ROS) concentrations at the root meristem. Upon exposure to metal stresses (e.g. toxic concentrations of cadmium (Cd) or copper (Cu), shown as brown circles), GPL4 induces excess ROS accumulation, leading to suppression of root growth in heavy metal‐contaminated soils, and thereby allows colonization of root growth in noncontaminated regions. Discontinuation of root growth in Cd‐contaminated soil reduces root exposure to the toxic metals and consequently reduces heavy metal accumulation in the aerial parts. The intensity of the red color at the root tips in the diagram depicts the concentration of ROS, with high concentrations being the most intense. RAM, root apical meristem.

Being sessile organisms, plants avoid stresses by redirecting their growth or changing their morphological development (Potters *et al*., 2007). For example, when a root encounters heavy metals in soil, it grows away from them. In this study, we found that a transcription factor, GPL4, is an important component of the heavy metal avoidance response (Fig. 7).

### GPL4 is required for the root avoidance response to toxic metals

Localized cadmium (Cd) treatment is known to inhibit primary root growth (avoidance) and enhance lateral root formation (colonization) and thereby promote exploration of noncontaminated soil (Remans *et al*., 2012). Our split media assays clearly showed that GPL4 suppressed plants by chimeric repressor gene silencing technology (CRES‐T) or RNA interference (RNAi) failed to avoid Cd contamination, whereas the wild‐type (WT) and overexpressing GPL4 (OX) plants clearly exhibited the avoidance response (Figs 3, 4, S4; Tables S2–S4). It seems likely that the CRES‐T and RNAi plants were unable to grow more roots in the control medium, because growth continued in the Cd‐containing medium. Thus, plants lacking GPL4 failed to re‐allocate biomass in response to localized Cd treatment, which is a necessary step for the avoidance response and for foraging to find a better medium to grow (Hodge, 2004, 2006, 2009). Moreover, CRES‐T and RNAi plants accumulated two to three times more Cd in the shoot than did the WT in the split media experiment (Fig. 3d,g), probably because more Cd was taken up by roots growing in the side containing Cd compared with the WT. However, shoot growth of the CRES‐T and RNAi lines was not affected by the high accumulation of Cd (Figs 3, 4), at least not within 3 wk of sowing. Together, our data suggest that GPL4 inhibits root growth in Cd‐contaminated soil patches, thereby reducing exposure of the root to Cd and limiting Cd accumulation in the shoot.

The critical role of GPL4 in root avoidance is not limited to Cd stress, but is also required for responses to other metal stresses. Copper (Cu) and zinc (Zn) are essential metals, but become toxic to plants in excess (Sharma *et al*., 2004; D'Souza & Devaraj, 2012; Saha *et al*., 2012). GPL4 CRES‐T and RNAi plants exhibited better root growth in the presence of excess Cu, Zn and deficient Zn, compared with the WT, whereas OX plants exhibited the opposite (Fig. S9a–e). Moreover, GPL4 was induced upon exposure to Cu and Zn (Fig. S9f), suggesting that GPL4 is required for root avoidance to essential metals toxicity. The wild type clearly exhibited inhibition of root growth in chambers of excess Cu, Zn or deficient Zn, and root colonization in control chambers lacking metal stresses (Fig. 6). However, CRES‐T plants lacked such a response (Fig. 6; Table S8). The GPL4 function for avoidance response seemed to be specific to metal stresses, because CRES‐T lines exhibited root avoidance to salt stress similar to the WT (Fig. 6e–h). Thus, GPL4 seemed to contribute to the plant's avoidance response to metal stresses in the rhizosphere, but not to general abiotic stresses. Future studies should examine how GPL4 mediates metal‐related stress responses specifically.

In contrast to *A. thaliana*, a nonhyperaccumulating plant, metal hyperaccumulating plants, such as *Noccaea caerulescens* (formerly *Thlaspi caerulescence*) and *Sedum alfredii*, actively proliferate roots into soil patches containing high concentrations of Cd and Zn (Whiting *et al*., 2000; Liu *et al*., 2010). This phenomenon was proposed to be due to their high internal requirements for these metals (Whiting *et al*., 2000; Liu *et al*., 2010). An interesting future study would be to examine whether such hyperaccumulator plants have reduced expression of homologs of *AtGPL4*.

### GPL4 regulates root apical meristem activity under normal and Cd stress conditions

Our data indicated that GPL4 is a transcription factor that regulates root growth (Figs 1, S6) by modulating the meristem activity (Fig. 5). Suppression of GPL4 function (CRES‐T) and *GPL4* expression (RNAi) resulted in longer roots (Figs 1a,b, S6) and more cells at the root apical meristem (RAM) (Fig. 5f,g) and overexpression of GPL4 (OX) had the opposite effect (Figs 1, 5, S6) in Cd stress condition. However, the role of GPL4 in the control of RAM size does not seem to be exclusive to Cd stress conditions, because the roots of the CRES‐T and RNAi lines were slightly longer than those of the WT at early seedling growth, even under normal conditions (Figs 5g, S6). Thus, we speculate that, under normal conditions, GPL4 plays a role in maintaining a suitable RAM size and upon Cd stress, GPL4 functions as a Cd‐avoidance factor, dramatically reducing root growth in Cd‐containing regions of medium (Figs 3, 4, S4). However, under normal condition (Fig. S6), the difference in root length between genotypes was smaller than under metal stress conditions (Fig. 1). Thus, GPL4 seems to play a more important role in stress response than under normal conditions. Cd‐mediated induction of *GPL4* at both transcript and protein levels supports our idea (Fig. 2).

### GPL4 inhibits root growth by activating reactive oxygen species production

Reactive oxygen species (ROS) are secondary messengers that mediate the developmental reprogramming of roots under stress conditions (Bailey‐Serres & Mittler, 2006; Swanson & Gilroy, 2010; Huang *et al*., 2012; Remans *et al*., 2012; Ditengou *et al*., 2015) and determine root meristem activity (Alfonso *et al*., 2009; Tsukagoshi *et al*., 2010). Five lines of evidence suggest that GPL4 regulates ROS concentrations at the root tip and that ROS generation is a major mechanism of GPL4‐mediated root growth inhibition in response to Cd. First, suppression of GPL4 (CRES‐T and RNAi) reduced ROS concentrations in root tips, whereas OX considerably increased it, both in the absence and presence of Cd (Fig. 5c–e). Second, the response of GPL4 CRES‐T plants to the ROS inducer PQ was less sensitive than that of the WT (Fig. S7), as was the response of these plants to Cd (Fig. 3). Third, a ROS quencher, KI, cancelled the hypersensitive response of OX lines to Cd (Fig. S7d,e); the root length of OX lines no longer differed from that of the WT when the medium was supplemented with KI, in addition to Cd. Fourth, transcriptome analysis revealed that the expression levels of many genes related to oxidative stress are altered in CRES‐T lines (Figs 5a,b, S3d; Table S6), including several peroxidases, which play important roles in ROS generation (Kawano, 2003; Kim *et al*., 2010a,b). Finally, the downstream targets of GPL4 are involved in ROS generation at root tips in response to Cd stress (Fig. S5d).

We identified two potential target genes of GPL4, ORPC4 and ERF61 (Fig. S5). ORP4C is a member of the oxysterol‐binding protein (OSBP)‐related protein family. OSBPs are involved in sterol trafficking and homeostasis (Saravanan *et al*., 2009; Raychaudhuri & Prinz, 2010; Olkkonen & Li, 2013; Barajas *et al*., 2014), and Cd and Cu perturb sterol homeostasis (Jones *et al*., 1987; Hernández & Cooke, 1997; Riches *et al*., 1998), which in turn triggers ROS production (Posé *et al*., 2009; Bonneau *et al*., 2010; Kim *et al*., 2010a,b). Therefore, we speculate that GPL4 alters sterol homeostasis by activating ORP4C, and thereby causes ROS generation. Previous reports indicate that many members of the Ethylene Responsive Transcription Factor family are involved in the plant's response to abiotic stress (Dubouzet *et al*., 2003; Gao *et al*., 2008; Wu *et al*., 2008) and modulate ROS concentrations and oxidative stress (Tang *et al*., 2005; Kim *et al*., 2012) by regulating ROS scavengers (Shaikhali *et al*., 2008). Therefore, GPL4 most likely inhibits root growth by activating the expression of genes required for ROS generation. Other possibilities such as regulation of metal transport or chelation processes are much less likely, as shown in Fig. S8.

CRES‐T and RNAi shoots seem to endure higher concentrations of Cd than do WT and OX shoots (Figs 1, 3, 4); they were much healthier in the presence of Cd (Fig. 1) and exhibited no apparent toxicity symptoms even when accumulated high concentrations of Cd in the split media assays (Figs 3, 4). This is most likely due to ectopic effects of CRES‐T and RNAi, which might reduce Cd‐induced ROS concentrations in the shoots of the CRES‐T and RNAi plants (Fig. 5e). Thus, CRES‐T and RNAi of GPL4 might have prevented excessive ROS generation and consequently oxidative damage due to Cd toxicity (Hossain *et al*., 2012). Moreover, Cd tolerance mechanisms involving Cd transport and glutathione‐dependent Cd chelation (Hossain *et al*., 2012) did not seem to be regulated by GPL4 (Fig. S8). Similarly, the ectopic generation of ROS in OX lines (Fig. 5c–e) may explain why root avoidance was not greater in these lines than in the WT (Table S2).

Changes in plant morphology are often the outcome of complex interactions of hormones, with auxin usually playing the central role (Overvoorde *et al*., 2010). We postulate that auxin function is critical for the morphological changes associated with root avoidance of Cd, too. The distribution and homeostasis of auxin is profoundly affected by Cd stress (Potters *et al*., 2007; Vitti *et al*., 2014; Wang *et al*., 2015), which can explain the Cd‐induced inhibition of primary root growth and increase of lateral root development. Future studies are needed to determine whether GPL4 indeed regulates Cd‐induced changes in auxin homeostasis and distribution.

### Potential of GPL4 as a tool to increase phytoextraction of heavy metals

Heavy metal hyperaccumulating plants, such as *Noccaea caerulescens* (formerly *Thlaspi caerulescence*), *Noccaea praecox* and *Arabis paniculata*, have received much attention as potential candidates for phytoextraction of heavy metals from contaminated soils. However, their slow growth and small biomass remain major obstacles for applications in phytoextraction (Meyer & Verbruggen, 2012; Souza *et al*., 2013). Therefore, using genetic engineering techniques to convert nonhyperaccumulators with large biomasses and rapid growth rates into hyperaccumulators presents an attractive strategy for cleaning up contaminated soils. To become a hyperaccumulator, a plant needs to acquire the capacity to penetrate and exploit contaminated soils with its roots, mobilize heavy metals from soil particles and rapidly translocate heavy metals from the root to the shoot. Previous efforts to enhance phytoextraction capacity sought to introduce genes encoding enzymes that synthesize chelators of heavy metals (Guo *et al*., 2012) or transporters that sequester heavy metals into vacuoles (Shim *et al*., 2013). In this study, we demonstrate another way to increase phytoextraction capability: manipulating the roots of nonhyperaccumulators to continue to grow in contaminated soils. *Arabidopsis thaliana* plants with suppressed GPL4 function or expression could proliferate roots in Cd‐contaminated environments, thereby accumulating two‐ to three‐fold more Cd in the aerial parts than the WT (Figs 3, 4, S4). These results suggest that genetic manipulation of GPL4 or its equivalent gene can effectively increase the phytoextraction capacity of nonhyperaccumulator plants, particularly if combined with other Cd tolerance and sequestration genes, such as *AtABCC1* and *AtABCC2*, which encode transporters that sequester Cd into vacuoles (Park *et al*., 2012).

## Author contributions

D.K., J‐U.H. and Y.L. designed the research; D.K. screened for Cd‐tolerant CRES‐T lines and performed the functional characterization of the GPL4 transcription factor; S.L. and D.H. analysed the transcriptome data; N.M. and M.O‐T. generated CRES‐T lines; W‐Y.S. measured metal contents; and D.K., J‐U.H., E.M. and Y.L. wrote the article.

## Supporting information

Please note: Wiley Blackwell are not responsible for the content or functionality of any Supporting Information supplied by the authors. Any queries (other than missing material) should be directed to the *New Phytologist* Central Office.

**Fig. S1** Cd inhibits the growth of *Arabidopsis thaliana* plants (related to Figs 1, 3 and 4).
**Fig. S2** *GPL4* RNAi specifically suppresses *GPL4* expression in *Arabidopsis thaliana* (related to Fig. 1).
**Fig. S3** GPL4 expression pattern in *Arabidopsis thaliana* and transcriptional activation activity in *A. thaliana* and yeast (related to Figs 2 and 5).
**Fig. S4 **GPL4‐dependent root biomass reallocation in *Arabidopsis thaliana* in response to Cd toxicity, in vertical split medium (related to Fig. 3).
**Fig. S5** GPL4 transcriptionally regulates genes involved in oxidative stress in *Arabidopsis thaliana* (related to Fig. 5).
**Fig. S6** Root growth of the wild‐type and GPL4 transgenic *Arabidopsis thaliana* plants (related to Fig. 5).
**Fig. S7** GPL4 regulates root growth by ROS generation in *Arabidopsis thaliana* (related to Fig. 5).
**Fig. S8** GPL4 transgenic *Arabidopsis thaliana* lines did not differ in metal contents and growth in the presence of a glutathione biosynthesis inhibitor.
**Fig. S9** GPL4 is important for normal responses to Cu or Zn excess and Zn deficiency in *Arabidopsis thaliana* (related to Fig. 6).
**Table S1** Summary of primers used in this study
**Table S2** Growth of wild‐type and GPL4 transgenic plants in the vertical split 0.5×MS agar medium assays presented in Figs 3 and S4
**Table S3** Growth of wild‐type and GPL4 CRES‐T plants in the vertical split soil assay presented in Fig. 3
**Table S4** Growth of the wild‐type and GPL4 CRES‐T plants in the horizontal split media assay presented in Fig. 4
**Table S5** Gene ontology biological processes represented by the genes in Clusters 1–5
**Table S6** DEGs related to oxidative stress
**Table S7** Growth of wild‐type and CRES‐T plants in the vertical split media assays shown in Fig. S7
**Table S8** Growth of wild‐type and CRES‐T plants in the vertical split media assays shown in Fig. 6
**Methods S1** Analysis of transcriptional activation activity and promoter binding assay.
**Methods S2 **Yeast one‐hybrid (Y1H) analysis.
**Notes S1** Accession numbers.
