# Supplementary material for: Root avoidance of toxic metals requires the GeBP‐LIKE 4 transcription factor in Arabidopsis thaliana
Source: New Phytol. 2016 Oct 21;213(3):1257–73. doi: 10.1111/nph.14242 (PMC5248625; doi:10.1111/nph.14242)
Supplement: Supplementary file 1 — Fig. S1 Cd inhibits the growth of Arabidopsis thaliana plants (related to Figs 1, 3 and 4). Fig. S2 GPL4 RNAi specifically suppresses GPL4 expression in Arabidopsis thaliana (related to Fig. 1). Fig. S3 GPL4 expression pattern in Arabidopsis thaliana and transcriptional activation activity in A. thaliana and yeast (related to Figs 2 and 5). Fig. S4 GPL4‐dependent root biomass reallocation in Arabidopsis thaliana in response to Cd toxicity, in vertical split medium (related to Fig. 3). Fig. S5 GPL4 transcriptionally regulates genes involved in oxidative stress in Arabidopsis thaliana (related to Fig. 5). Fig. S6 Root growth of the wild‐type and GPL4 transgenic Arabidopsis thaliana plants (related to Fig. 5). Fig. S7 GPL4 regulates root growth by ROS generation in Arabidopsis thaliana (related to Fig. 5). Fig. S8 GPL4 transgenic Arabidopsis thaliana lines did not differ in metal contents and growth in the presence of a glutathione biosynthesis inhibitor. Fig. S9 GPL4 is important for normal responses to Cu or Zn excess and Zn deficiency in Arabidopsis thaliana (related to Fig. 6). Table S1 Summary of primers used in this study Table S2 Growth of wild‐type and GPL4 transgenic plants in the vertical split 0.5×MS agar medium assays presented in Figs 3 and S4 Table S3 Growth of wild‐type and GPL4 CRES‐T plants in the vertical split soil assay presented in Fig. 3 Table S4 Growth of the wild‐type and GPL4 CRES‐T plants in the horizontal split media assay presented in Fig. 4 Table S5 Gene ontology biological processes represented by the genes in Clusters 1–5 Table S6 DEGs related to oxidative stress Table S7 Growth of wild‐type and CRES‐T plants in the vertical split media assays shown in Fig. S7 Table S8 Growth of wild‐type and CRES‐T plants in the vertical split media assays shown in Fig. 6 Methods S1 Analysis of transcriptional activation activity and promoter binding assay. Methods S2 Yeast one‐hybrid (Y1H) analysis. Notes S1 Accession numbers. [file NPH-213-1257-s001.pdf]

New Phytologist

***New Phytologist* Supporting Information Figs S1–S9, Tables S1–S4, S6 & S7, Methods S1 & S2 and Notes S1**

Article title: Root avoidance of toxic metals requires the GeBP-Like 4 transcription factor in *Arabidopsis thaliana*

Authors: Deepa Khare, Nobukata Mitsuda, Seungchul Lee, Won-Yong Song, Daehee Hwang, Masaru Ohme-Takagi, Enrico Martinoia, Youngsook Lee and Jae-Ung Hwang

Article acceptance date: 30 August 2016

The following Supporting Information is available for this article:

Fig. S1 Cd inhibits the growth of *Arabidopsis thaliana* plants (related to Figs 1, 3, and 4).

Fig. S2 *GPL4* RNAi specifically suppresses *GPL4* expression in *Arabidopsis thaliana* (related to Fig. 1).

Fig. S3 GPL4 expression pattern in *Arabidopsis thaliana* and transcriptional activation activity in *A. thaliana* and yeast (related to Figs 2 and 5).

Fig. S4 GPL4-dependent root biomass re-allocation in *Arabidopsis thaliana* in response to Cd toxicity, in vertical split medium (related to Fig. 3).

Fig. S5 GPL4 transcriptionally regulates genes involved in oxidative stress in *Arabidopsis thaliana* (related to Fig. 5).

Fig. S6 Root growth of the wild-type and GPL4 transgenic *Arabidopsis thaliana* plants (related to Fig. 5).

Fig. S7 GPL4 regulates root growth by ROS generation in *Arabidopsis thaliana* (related to Fig. 5).

Fig. S8 GPL4 transgenic *Arabidopsis thaliana* lines did not differ in metal contents and growth in the presence of a glutathione biosynthesis inhibitor.

Fig. S9 GPL4 is important for normal responses to Cu or Zn excess and Zn deficiency in *Arabidopsis thaliana* (related to Fig. 6).

Table S1 Summary of primers used in this study

Table S2 Growth of wild-type and GPL4 transgenic plants in the vertical split ½ MS agar medium assays presented in Figs 3 and S4

Table S3 Growth of wild-type and GPL4 CRES-T plants in the vertical split soil assay presented in Fig. 3

Table S4 Growth of the wild-type and GPL4 CRES-T plants in the horizontal split media assay presented in Fig. 4

Table S5 Gene ontology biological processes represented by the genes in Clusters 1–5

Table S6 DEGs related to oxidative stress

Table S7 Growth of wild-type and CRES-T plants in the vertical split media assays shown in Fig. S7

Table S8 Growth of wild-type and CRES-T plants in the vertical split media assays shown in Fig. 6

Methods S1 Analysis of transcriptional activation activity and promoter binding assay.

Methods S2 Yeast one-hybrid (Y1H) analysis.

Notes S1 Accession numbers.

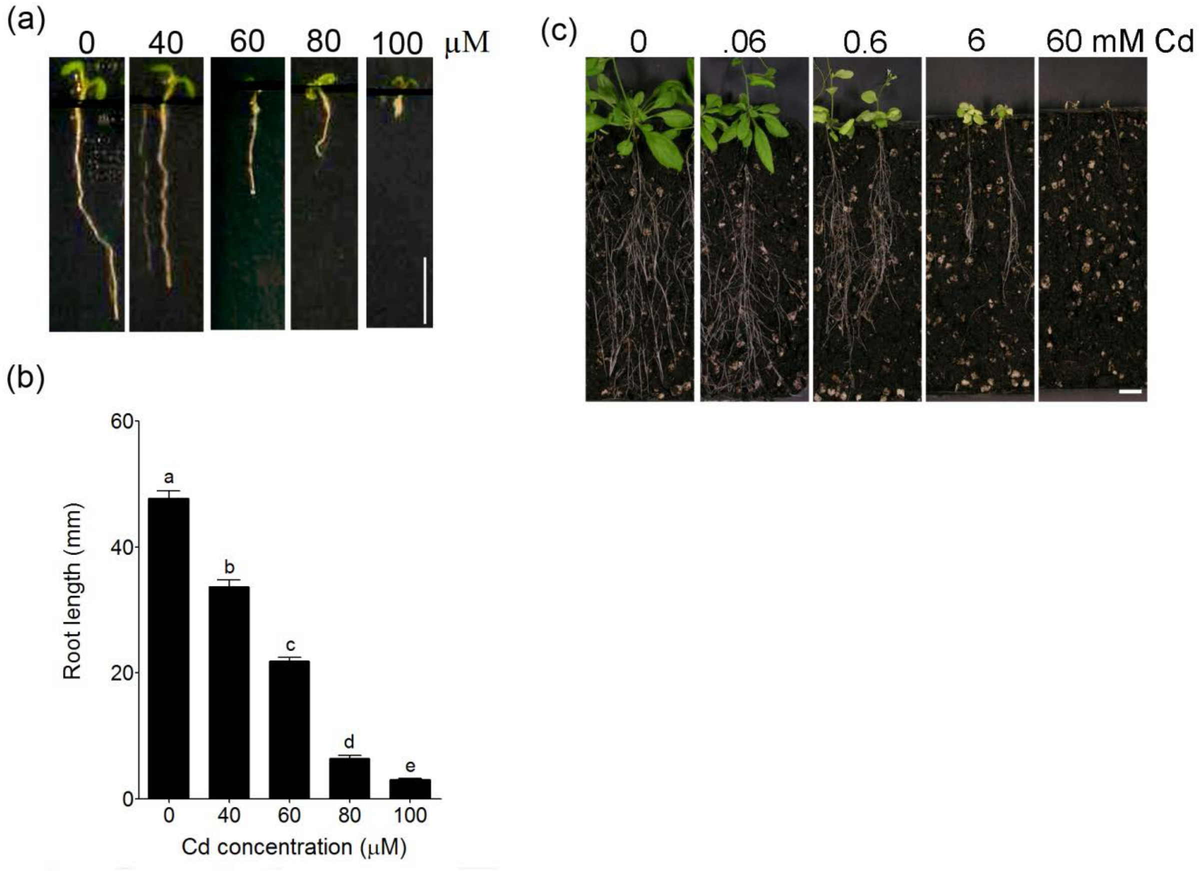

Fig. S1 Cd inhibits the growth of *Arabidopsis thaliana* plants (related to Figs 1, 3, and 4). (a, b) Wild-type seedlings after 1 wk of growth on ½ MS agar plates containing different concentrations of $CdCl_2$ (0, 40, 60, 80, and 100 μM) (a). Mean values (± SE) of root length of 25 seedlings for each concentration (b), and different letters represent a significant difference between concentrations (Tukey's test, $P < 0.01$). (c) Growth of wild-type plants at different concentrations of $CdCl_2$ (0, 0.06, 0.6, 6, and 60 mM) for 3 wk in rhizoboxes. Six seedlings tested for each concentration exhibited consistent dose-dependency. Bars, 1 cm.

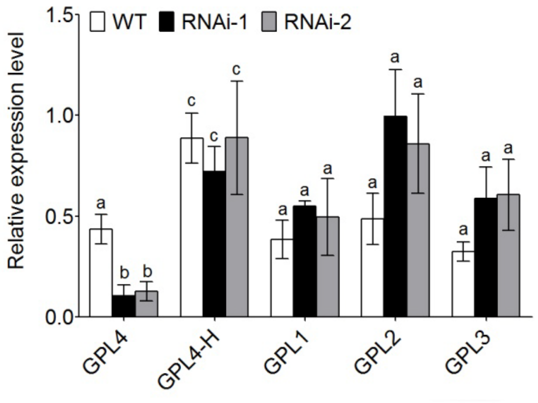

Fig. S2 *GPL4* RNAi specifically suppresses *GPL4* expression in *Arabidopsis thaliana* (related to Fig. 1). Relative expression levels of the closest *GPL4* homolog (*GPL4-H*) and other studied members (*GPL1, GPL2, GPL3*) of the GeBP family in GPL4 knockdown lines (RNAi). Transcript levels were normalized by *tubulin 8* as an internal control. Combined results (mean values ± SE) of three independent experiments are shown. Each experiment was performed with three replicates. Different letters represent a significant difference between WT and RNAi lines (Tukey's HSD test; $P < 0.01$).

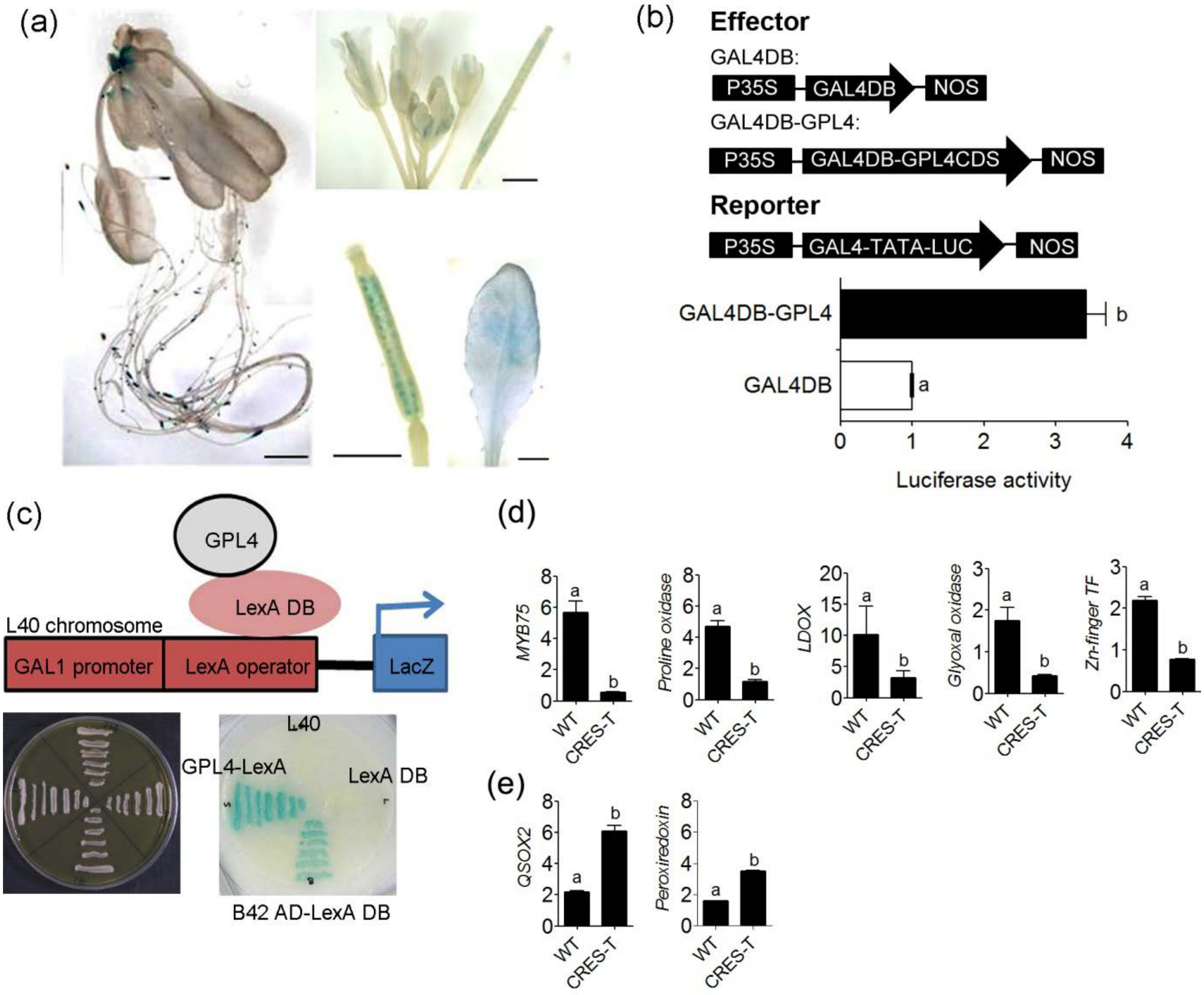

Fig. S3 GPL4 expression pattern in *Arabidopsis thaliana* and transcriptional activation activity in *A. thaliana* and yeast (related to Figs 2 and 5). (a) Tissue-specific expression of GPL4 in *GPL4$_{pro}$:GPL4:GUS* seedlings: 3-wk-old plants (left) and young flowering buds (right, top) , developing seeds (middle, bottom), and a senescing leaf (right, bottom) of mature plants. Bars: left, 1 cm; middle and right, 1 mm. (b) Transcriptional activation activity of GPL4 in *A. thaliana*. Schematic representation of effector and reporter constructs used to analyze GPL4 transcription activation activity using a transient expression assay in *A. thaliana* leaves (top). Effector plasmids expressed the GAL4 DNA-binding (GAL4DB) domain alone or fused to the *GPL4* coding sequence (*GPL4CDS*) driven by the 35S promoter. The reporter plasmid contained a minimal CaMV 35S promoter (P35S) upstream of the GAL4 binding site (GAL4) fused to TATA boxes and the luciferase reporter (LUC). Mean luciferase activity values (± SE) of three independent experiments with at least three replicates each (bottom). Different characters represent a significant difference between means analyzed by Tukey's HSD test (P < 0.05). (c) Scheme of transcriptional activation

activity assay in yeast (top). The L40 yeast strain, which harbors the *LacZ* reporter gene under the control of the GAL1 promoter and LexA operator, was transformed with *GPL4* fused with the *LexA* DNA-binding domain (LexA DB). Transcriptional activation activity of GPL4 observed using an X-gal filter lift assay (bottom). Yeast grown on YPDA medium (bottom left) and the result of the X-gal filter lift assay (bottom right). (d, e) Confirmation of gene expression changes in transcriptome analysis. Transcript levels of oxidative stress-related genes from Cluster 1 (d) and Cluster 3 (e) were assessed using qRT-PCR in wild-type and CRES-T plants 2 h after Cd treatment. Combined results (mean ± SE) of three independent experiments are shown. Each experiment was performed with at least three replicates. Different characters represent a significant difference between means analyzed by Tukey's HSD test ($P < 0.05$).

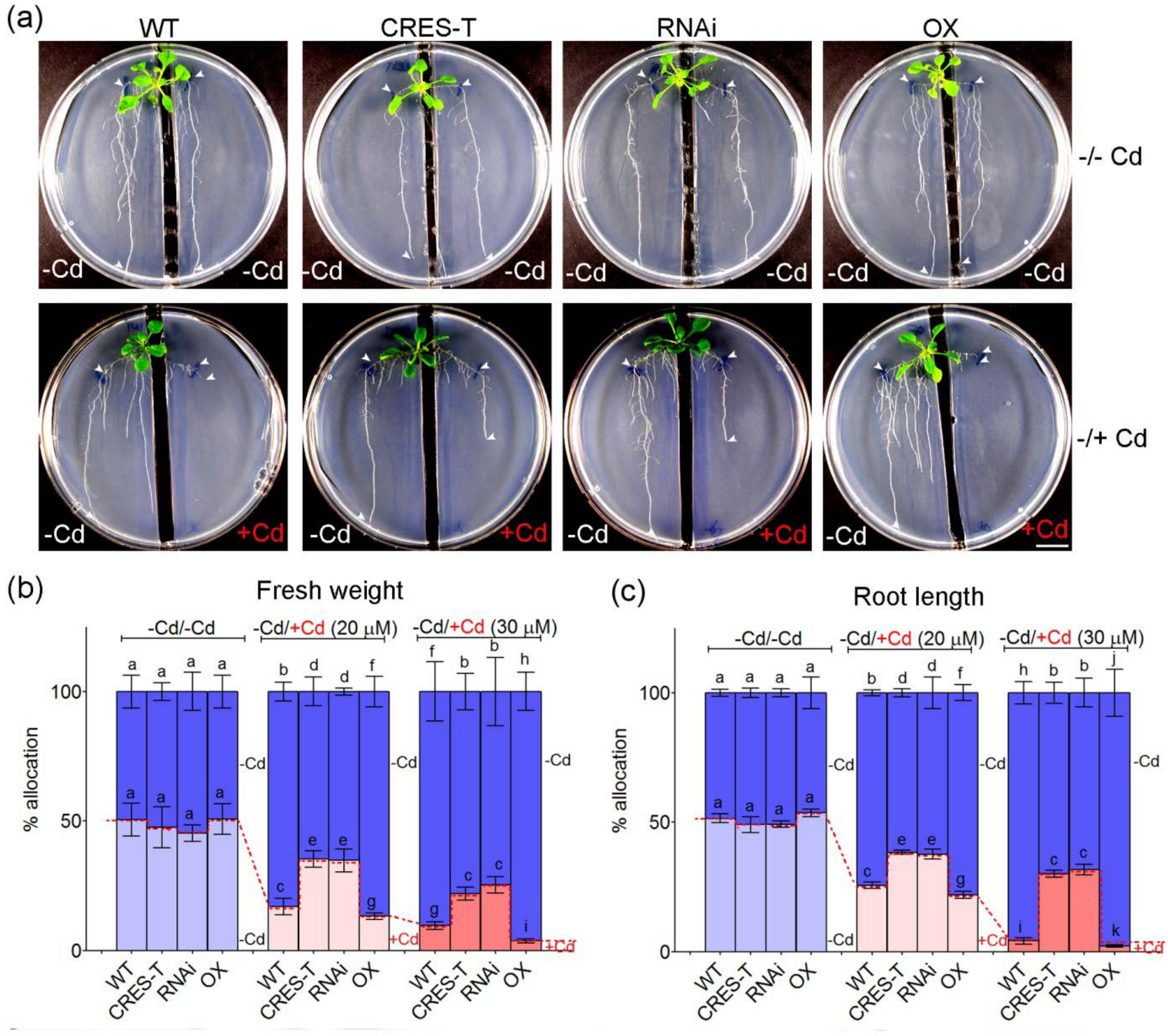

Fig. S4 GPL4-dependent root biomass re-allocation in *Arabidopsis thaliana* in response to Cd toxicity, in vertical split medium (related to Fig. 3). (a) Growth of wild-type (WT) and GPL4 transgenic seedlings in split media supplemented with water (-Cd) and 30 µM $CdCl_2$ (+Cd). Representative seedling images of homogeneous (-/-Cd; upper panel) with water supplementation at both sides, and heterogeneous Cd (-/+Cd; lower panels) supplementation. Arrowheads indicate net growth of the main roots after transfer. Bar, 1 cm. (b, c) Mean percentage allocation (± SE) of root fresh weight (b) and the longest root length (c) on the two sides of the split media for seedlings tested in (a). Combined results (mean ± SE) of three independent experiments are given. The percentage values in the left (-Cd) are in the upper segments (blue) of the bars and those in the right (-Cd for '-/-Cd'; light blue and +Cd for '-/+Cd'; pink, increased color intensity denotes an increase in Cd concentration) are in the lower segments. Each experiment was performed with greater than

or equal to six seedlings per genotype per treatment. Different letters indicate a significant difference in percentage allocation between genotypes or treatments (Tukey's HSD test, $P < 0.01$).

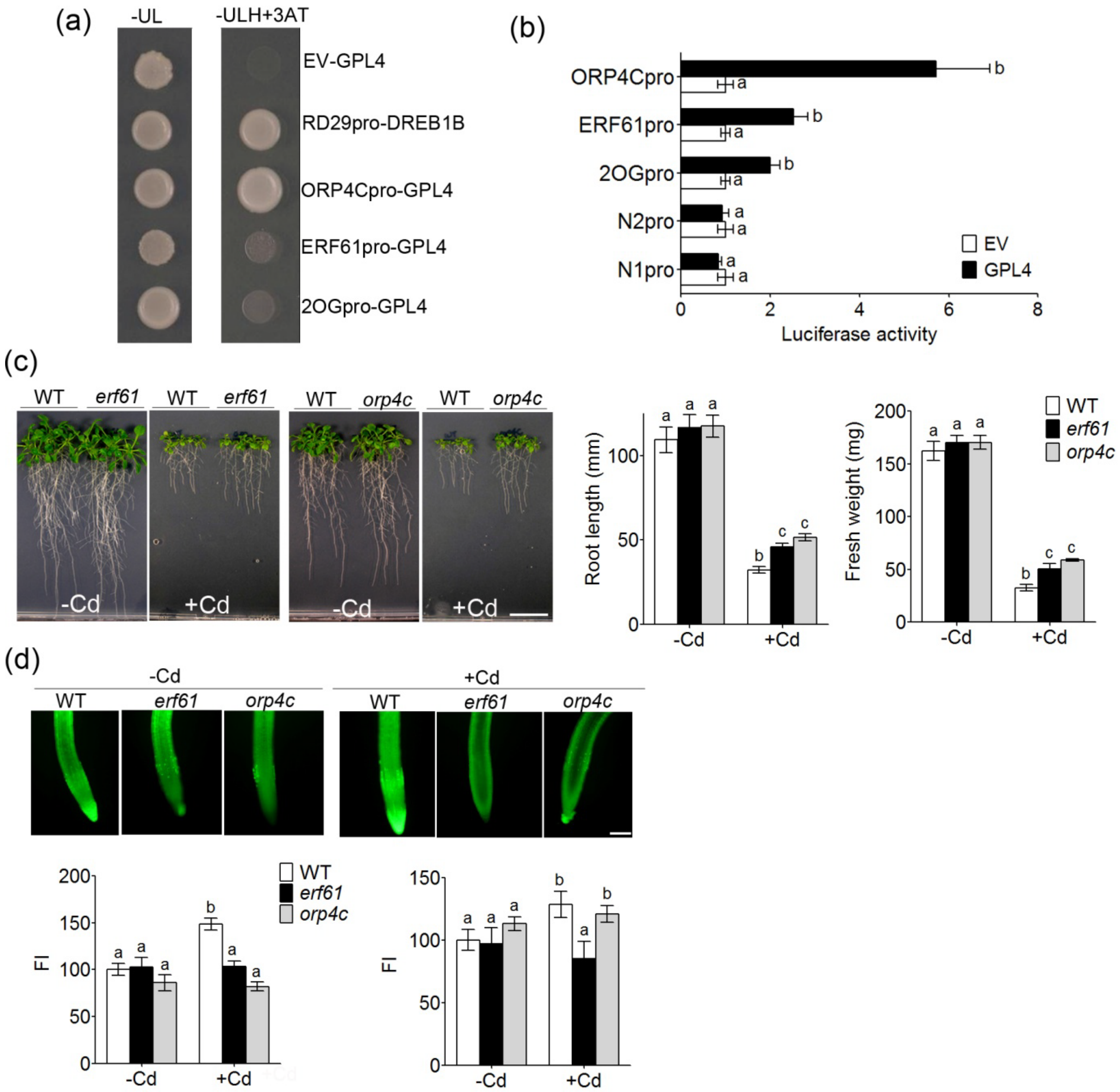

Fig. S5. GPL4 transcriptionally regulates genes involved in oxidative stress in *Arabidopsis thaliana* (related to Fig. 5). (a, b) Regulation of downstream target genes by GPL4. Interaction of GPL4 with the promoters of *ORP4C* (*ORP4Cpro*), *ERF61* (*ERF61pro*), and *2-oxoglutarate-Fe (II)-dependent dioxygenase* (*2OGpro*) demonstrated by an Y1H assay (a). The *RD29pro* and DREB1B interaction was used as a positive control. Empty vector (EV) and GPL4 were used as a negative control. Transactivation of a luciferase reporter by GPL4 through interaction with promoters of target genes in a transient assay in *A. thaliana* leaves (b). Relative values of luciferase activity to that of the empty vector control (EV, white bars) are presented. *N1pro* and *N2pro* are the promoters to which GPL4 did not bind in the Y1H assay, and GPL4 failed to enhance the luciferase activity through those promoters. Mean values (± SE) of three independent experiments, with three replicates each are shown. (c) Cd

tolerance of *erf61* and *orp4c* knockout mutants. Representative images of seedlings grown on ½ MS media without or with 70 µM $CdCl_2$ supplementation (-Cd and +Cd, respectively, top). Mean values (± SE) of root length (lower left) and fresh weight (lower right) from three independent experiments are shown. (d) ROS levels assessed using DCF fluorescence in *erf61* and *orp4c* mutants. DCF fluorescence of the root tips of *erf61* and *orp4c* mutants without and with Cd treatment (-Cd and +Cd, respectively, upper). Relative values of fluorescence intensity measured from the root tip images (bottom left, $n \geq 20$) and for whole seedlings of *erf61* and *orp4c* (bottom right, $n = 30$) compared to the wild-type control. Results are mean values (±SE) of three independent experiments with 10 seedlings per genotype and experiment. Different characters indicate a significant difference of means compared to the WT analyzed by Tukey's HSD test at $P \leq 0.05$ (b–d). Bars: (c) 1 cm; (d) 50 µm.

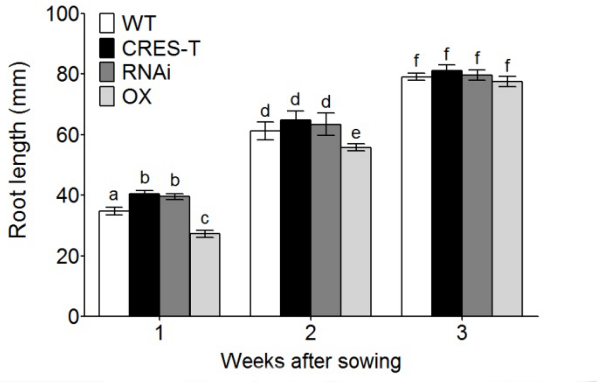

Fig. S6 Root growth of the wild-type (WT) and GPL4 transgenic *Arabidopsis thaliana* plants (related to Fig. 5). Root length measurement of the GPL4 transgenic lines (CRES-T, RNAi, and OX) grown on ½ MS media for 1, 2, and 3 wk after sowing. Seed germination was not different between genotypes. The difference in root length was significant only at the early stage of seedling growth (1–2 wk after sowing) under standard conditions. Mean values (± SE) of three independent experiments are shown. Each individual experiment was performed with 10 seedlings per time point per genotype. Different letters represent statistically significant differences between genotypes and time points analyzed by Tukey's HSD test ($P < 0.01$).

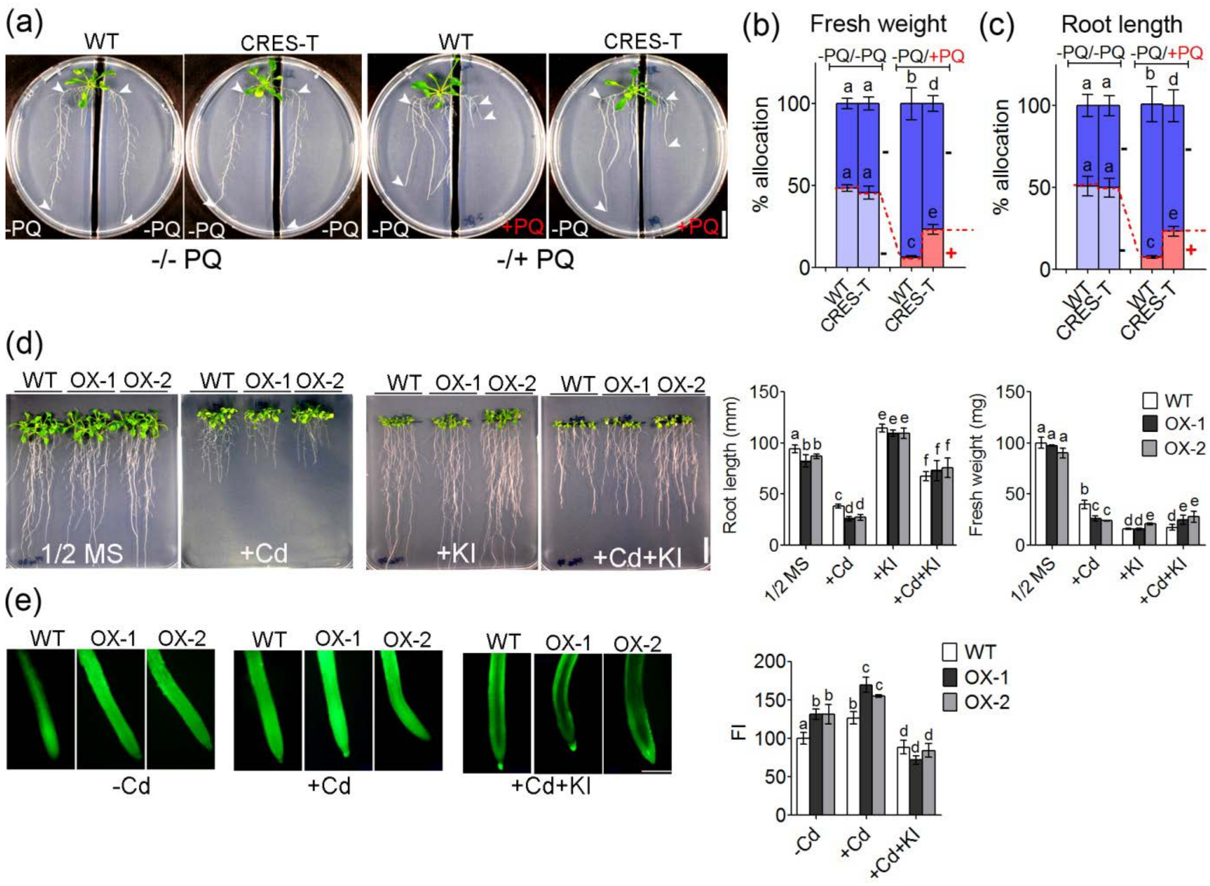

Fig. S7 GPL4 regulates root growth by ROS generation in *Arabidopsis thaliana* (related to Fig. 5). (a) Growth of wild-type (WT) and CRES-T seedlings in vertically split media supplemented with water (-PQ; paraquat) and 5 nM PQ (+PQ). Left panel is the homogeneous (-/- PQ) no treatment control with water supplementation on two sides, and the right panel shows heterogeneous PQ (-/+PQ) supplementation in the right side of the medium. Arrowheads indicate net growth of the main roots after transfer. Representative images from 10 seedlings per genotype per treatment are shown. Bar, 1 cm. (b, c) Mean percentage allocation (± SE) of root fresh weight (b) and the longest root lengths (c) of plants grown in the split media. The percentage values in the left (-PQ) are in the upper segments (blue) of the bars and those in the right (-PQ for '-/-PQ'; light blue and +PQ (5 nM) for '-/+PQ'; pink) are in the lower segments. (d) Growth of OX lines in the presence of ROS quencher KI (1 mM). Five seeds per genotype were sown and grown for 2 wk on a ½ MS agar plate without or with supplementation with 70 µM $CdCl_2$ (-Cd and +Cd, respectively, left) and with supplementation of KI in the presence or absence of Cd (right). The experiment was repeated three times, and combined results (mean ± SE) of total fresh weight (bottom left) and the longest root length (bottom right) are given. Each experiment was performed

with three plates per treatment and supported the same conclusion. Shoot growth, and consequently the total fresh weight, much reduced by KI, both in the wild type and OX lines. The extreme sensitivity of shoot growth to KI may be due to the critical role of ROS in cell expansion in the shoot (Lu *et al*., 2013). (e) ROS level assessment using H_2DCF-DA dye. No Cd control (-Cd), treated with 70 µM $CdCl_2$ (+Cd), and treated with 70 µM $CdCl_2$ and 1 mM KI together (+Cd+KI). Representative root tip images from three independent experiments are shown. Bar, 50 µm. Mean values (± SE) of relative DCF fluorescence intensity (FI) measured in root tip images. Each individual experiment was performed with at least six seedlings per genotype per treatment and supported the same conclusion. Different letters indicate a significant difference in percentage allocation and means between genotypes or treatments analyzed by Tukey's HSD test at $P < 0.05$ (b–e).

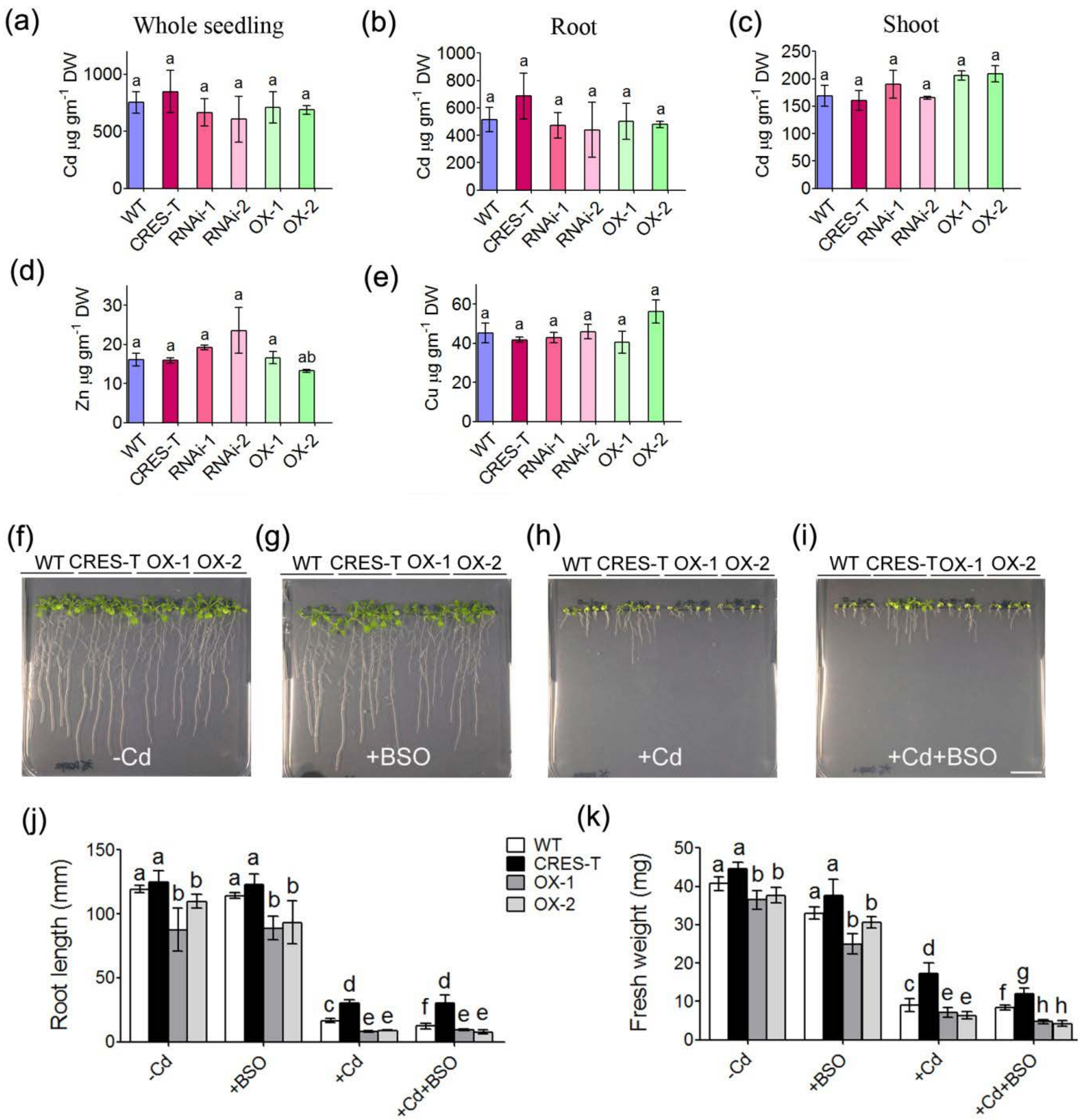

Fig. S8 GPL4 transgenic *A. thaliana* lines did not differ in metal contents and growth in the presence of a glutathione biosynthesis inhibitor. (a–e) Heavy metal contents of 2-wk-old wild-type (WT) and GPL4 transgenic (CRES-T, RNAi, and OX) seedlings grown on ½ MS medium supplemented with 10 µM $CdCl_2$. Mean values (± SE) of Cd contents in (a) whole seedlings, (b) roots, and (c) shoots and (d) Zn and (e) Cu contents in whole seedlings from three independent experiments. Each individual experiment was performed with 10 seedlings per genotype. (f–k) Effect of glutathione biosynthesis inhibitor, buthionine sulphoximine (BSO), on Cd tolerance of CRES-T and OX lines. Representative images of seedlings grown on control medium (f), medium containing BSO (+BSO; g), medium containing 70 µM $CdCl_2$ (+Cd; h), and medium containing both BSO (100 µg ml^{-1}) and Cd (+BSO +Cd; i).

Bar, 1 cm. Mean values (± SE) of the longest root length (j) and fresh weight per plate (k) of three independent experiments. Each individual experiment was performed with three plates (15 seedlings) per genotype per treatment and supported the same conclusion. Different letters indicate that means are significantly different between genotypes or treatments (Tukey's HSD (P < 0.05).

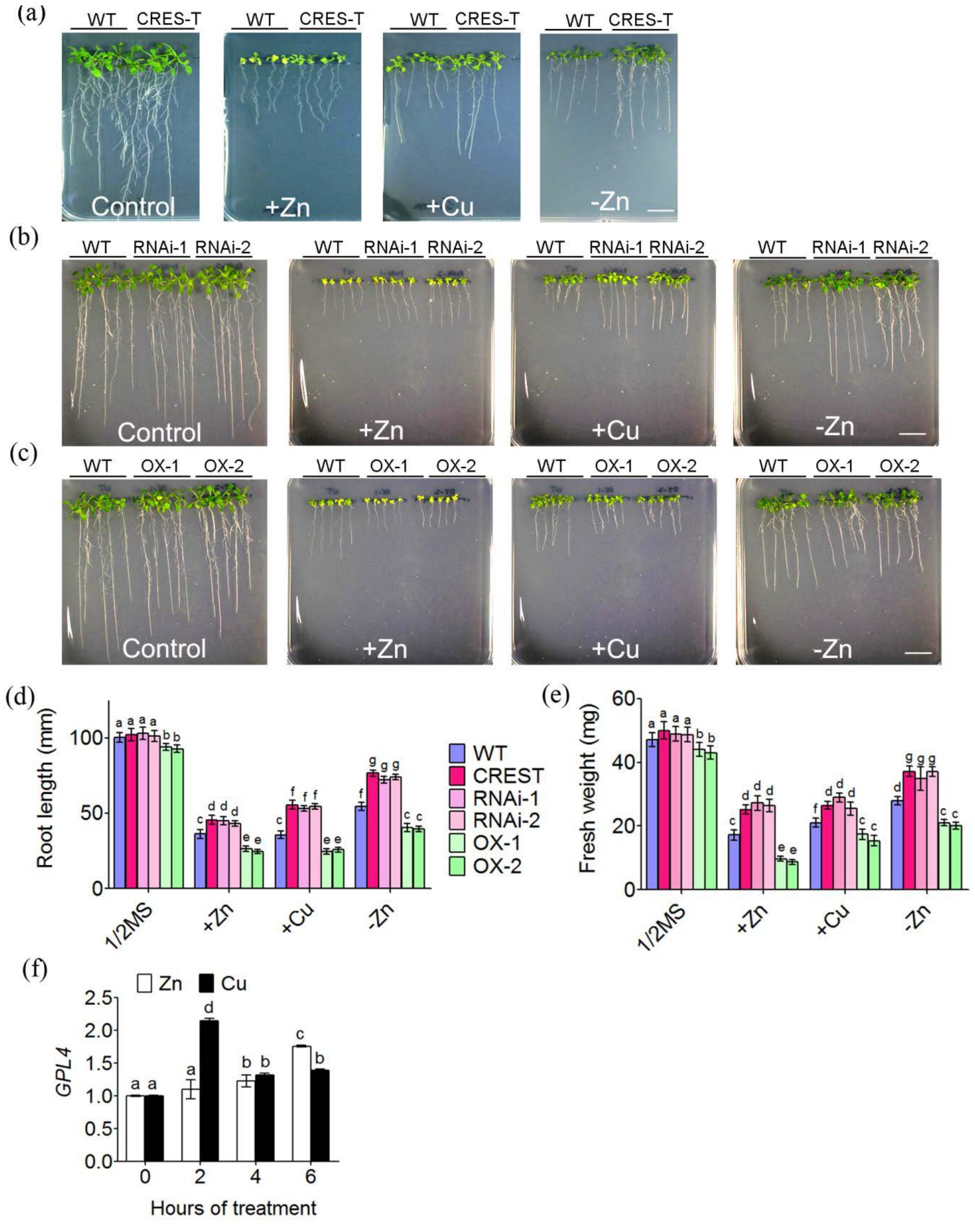

Fig. S9 GPL4 is important for normal responses to Cu or Zn excess and Zn deficiency in *Arabidopsis thaliana* (related to Fig. 6). (a–e) Growth comparison of wild-type (WT) and (a) CRES-T, (b) RNAi, and (c) OX plants in conditions of excess Zn (+Zn; 0.5 mM), excess Cu (+Cu, 65 μM), and Zn deficiency (-Zn with 5 μM of N, N, N', N'-Tetrakis-(2-pyridylmethyl) ethylenediamine; TPEN). Representative images from three independent experiments are shown. Bars, 1 cm. Combined results (mean ± SE) of (d) the longest root length and (e) total

fresh weight per plate are given. Each individual experiment was performed with three plates (15 seedlings) per genotype and treatment and supported the same conclusion. (f) *GPL4* transcript level changes after treatment with 65 µM Cu or 0.5 mM Zn. Combined results (mean ± SE) of three independent experiments are shown and each individual experiment was performed with three replicates per treatment and time point. Different letters indicate that the means are significantly different between time points or treatments analyzed by Tukey's HSD test ($P \leq 0.05$).

Table S1 Summary of primers used in this study

Purpose	Name	Primer sequence (5’-3’)
**GPL4 transgenic plants generation**		
CRES-T	35S-F	GAAGTTCATTTCATTTGGAGAGG
	SRDX-R	TTAAGCGAAACCCAAACGGAGTTCTAG
RNAi	Forward	CAGATAAGCCAATCGTCTTAACCAG
	Reverse	CTGTCTCAGCTTTGAAATCAATCATAC
OX	Forward	ATGAACAAGAAACTTCTCAACCCTCTTG
	Reverse	TTAGATAGTTTCAGCAATCACAGAAGCC
GUS, GFP	Forward	CAGAGGGTAAGTGGGATGCCCAAA
	Reverse	GATAGTTTCAGCAATCACAGAAGCCAC
**GPL4 transcriptional activity assay in yeast**	Forward	ATGAACAAGAAACTTCTCAACCCTCTTG
	Reverse	TTAGATAGTTTCAGCAATCACAGAAGCC
**Promoter-GPL4 binding assays**	AtERF61-F	CTGCTATCAAATGCGTGTTT
	AtERF61-R	CTCAACCAGACGTTTCGTGG
	2OG-Fe-F	GCAGTTTCTGTTGGTAAACC
	2OG-Fe-R	TCGGAGGATCTTTGTTTGATG
	AtORP4C-F	GGTAACGAGCGATGTTAGGT
	AtORP4C-R	TTCTTCTCCTTTTTTTCTGCG
	GPL4-F	ACGAAGCGAGCTAGTGAAGG
	GPL4-R	TCTCCTCCAAGAACCACCAC
**qRT-PCR**	MYB75- F	CGACTGCAACCATCTCAATG
	MYB75-R	TGTCCCCCTTTTCTGTTGTC
	GST-F	TTTCTTCGAACCGGTTTTTG
	GST-R	CCAAGACGGTCTATCCGAAA
	LDOX-F	TGGGTCACTGCAAAATGTGT
	LDOX-R	CGGAGACTCAACACTCACCA
	Peroxiredoxin-F	CGTCTCGTGCCCTTCATATT
	Peroxiredoxin-R	TCTTGAAACCCTGTGGGAAC
	QSOX-F	GGAACGATACCCTGGGAAAT
	QSOX-R	CGAAATCGCATATTGTCGTG
	NAT12-F	TCTTCCGGTCAGCTCTCATT
	NAT12-R	AACAGAACCAGGAGGTGGTG
	CAT3-F	CATCCACGCTCTTCTCAACA
	CAT3-R	GCCAAGGCCATTATCAGTGT
	TRX-F	CAGGGGACTATGCTGGAAAA
	TRX-R	GACTCTTTCAATGGCGGAAA
	ZFT-F	AATTTTGACCCGGAAAAACC
	ZFT-R	TACTTGGAGAAGCCGAGGAA
	GO-F	CGATGGAAGACGGAGATGAT
	GO-R	GGGCTTGAGCTGTCTGAAAC
	PO-F	GTTCTGGTTTCGGTGTCGTT
	PO-R	TTTAACCCGAAGGACAATGC

Table S2 Growth of wild-type and GPL4 transgenic plants in the vertical split ½ MS agar medium assays presented in Figs 3 and S4

Line	Control		Cd (10 µM)		Cd (30 µM)	
	L	R	L (-Cd)	R (+Cd)	L (-Cd)	R (+Cd)
Root fresh weight (mg)						
WT	5.3 ± 0.7[a]	5.4 ± 0.7[a]	6.5 ± 0.5[b]	2.5 ± 0.5[c]	6.9 ± 0.7[f]	0.6 ± 0.1[g]
CRES-T	5.8 ± 0.3[a]	5.3 ± 0.7[a]	3.7 ± 0.4[d]	3.9 ± 0.3[d]	4.6 ± 0.4[d]	1.3 ± 0.1[i]
RNAi	5.8 ± 0.6[a]	5.0 ± 0.2[a]	4.3 ± 0.1[d]	3.9 ± 0.2[d]	4.4 ± 0.7[d]	1.4 ± 0.1[i]
OX	5.0 ± 0.5[a]	5.2 ± 0.5[a]	6.2 ± 0.6[b]	2.0 ± 0.1[e]	6.7 ± 0.4[f]	0.2 ± 0.04[j]
Root length (mm)						
WT	147 ± 4.1[a]	156 ± 5.2[a]	143 ± 1.3[b]	63 ± 1.7[d]	132 ± 6[c]	6 ± 1.6[h]
CRES-T	143 ± 4.8[a]	138 ± 8.6[a]	137 ± 3.4[c]	125 ± 7.7[e]	131 ± 7.6[c]	57 ± 2.5[i]
RNAi	150 ± 4.6[a]	146 ± 3.5[a]	134 ± 7.6[c]	138 ±10.4[e]	123 ± 10[c]	57 ± 3.7[i]
OX	139 ± 5.1[a]	150 ± 2.6[a]	143 ± 1.4[b]	42 ± 3.4[f]	112 ± 10[g]	3 ± 0.5[j]

Absolute values (mean ± SE) of root fresh weight and the longest root length measured in the split media assays shown in Fig 3 and S4. In heterogeneous split media, root growth was dramatically reduced in the sides containing Cd (blue color), and root biomass was significantly increased in sides lacking Cd (red color) by the avoidance response in the wild type and OX, compared with the counterparts in control split media. Such root biomass increase in the side lacking Cd is likely due to development of more roots, and not to root length. By contrast, CRES-T and RNAi failed to exhibit such an avoidance response. Different characters in the superscript indicate significant difference in means between genotypes and treatment by Tukey's HSD test ($P \leq 0.05$).

Table S3 Growth of wild-type and GPL4 transgenic plants in the vertical split soil assay presented in Fig. 3

Root length (mm)								
Line	Control		1 mM $CdCl_2$		3 mM $CdCl_2$		6 mM $CdCl_2$	
	L	R	L (-Cd)	R (+Cd)	L (-Cd)	R (+Cd)	L (-Cd)	R (+Cd)
WT	137.0 ± 7.2[a]	124.1 ± 8.6[a]	147.6 ± 13.8[b]	79.3 ± 16.2[d]	135.1 ± 0.9[a]	54.8 ± 8.5[e]	134.9 ± 6.3[a]	51.1 ± 4.7[e]
CRES-T	138.2 ± 10.7[a]	129.4 ± 10.4[a]	122.0 ± 8.6[a]	126.4 ± 11.9[a]	140.8 ± 14.7[a]	111.4 ± 11.6[f]	138.4 ± 4.1[a]	95.2 ± 18.5[f]

Absolute values (mean ± SE) of the longest root length measured in the split soil assays shown in Fig. 3 are presented. Root biomass was not analyzed due to technical problem with soil experiment. In heterogeneous split media, root growth was dramatically reduced in the sides containing Cd (blue), and root biomass was significantly increased in sides lacking Cd (red color) by the avoidance response in the wild type and, compared with the counterparts in control split media. Different characters in the superscript indicate significant difference in means between genotypes and two sides of the medium by Tukey's HSD test, $P \leq 0.05$.

Table S4 Growth of the wild-type and GPL4 CRES-T plants in the horizontal split media assay presented in Fig. 4

Line	Control		10 µM $CdCl_2$	
	Top	Bottom	Top (-Cd)	Bottom (+Cd)
Root fresh weight (mg)				
WT	0.5 ± 0.1[a]	1.0 ± 0.6[b]	0.9 ± 0.1[d]	0.4 ± 0.03[e]
CRES-T	0.7 ± 0.1[a]	1.3± 0.1[c]	0.5 ± 0.1[a]	1.2 ± 0.1[c]
Lateral root numbers				
WT	3.9 ± 0.2[a]	8.2 ± 0.4[b]	5.6 ± 0.2 [d]	3.9 ± 0.2[a]
CRES-T	5.08 ± 0.3[c]	9.9 ± 0.4[d]	3.8 ± 0.2 [a]	8 ± 0.5[b]

Line	Control		70 µM $CdCl_2$	
	Top	Bottom	Top (-Cd)	Bottom (+Cd)
Root fresh weight (mg)				
WT	3.1 ± 0.1[a]	2.3 ± 0.2[b]	3.3±0.1[c]	0.13±0.01[e]
CRES-T	2.2 ± 0.1[b]	2.1± 0.1[b]	1.05 ± 0.1[d]	0.78 ± 0.04[f]
Lateral root numbers				
WT	14.06 ±0.3[a]	19.8 ± 0.8[b]	18 ± 0.3[d]	4.1 ± 0.3[e]
CRES-T	10.8 ± 0.6[c]	14 ± 0.6[a]	9.7 ± 0.3[c]	7.05 ± 0.3[f]

Absolute values (mean ± SE) of root fresh weight and lateral root number measured in the split root assays shown in Fig. 4. In heterogeneous split media, root biomass and lateral root number were dramatically reduced in the sides containing Cd (blue), whereas they were significantly increased in sides lacking Cd (red color) by the avoidance response in the wild type, compared with the counterparts in control split media. By contrast, such an avoidance response was significantly suppressed in CRES-T. Different characters in the superscript indicates significant difference in means between genotype and two sides of the medium analyzed by Tukey's HSD test, $P \leq 0.05$.

Table S6 DEGs related to oxidative stress

Gene AGI number	Annotation	Fold change (log_2 value) 0 h	2 h	*P*-value
**Cluster 1 genes**				
AT3G50700	Zn finger TF	0.1	-1.0	0.021
AT5G19580	Glyoxal oxidase	0.1	-0.90	0.003
AT1G64380	ERF/AP2 family TF (ERF61)	0.1	-1.1	0.01
AT3G30775	Proline oxidase	0.07	-1.2	0.01
AT5G54000	2OG-Fe(II)-dependent oxygenase	-0.1	-1.8	0.005
AT5G57240	ORP4C	0.2	-1.0	0.03
AT1G56650	MYB75	0.3	-1.0	0.003
AT5G17220	GST1	-0.001	-1.0	0.003
AT4G22880	Leucoanthocyanidin dioxygenase (LDOX)	0.03	-0.93	0.004
AT3G50990	Peroxidase 36 precursor	-0.04	-1.2	0.01
**Cluster 2 genes**				
AT1G20620	Catalase 3	-0.6	0.1	0.02
AT2G39040	Peroxidase 24	-0.8	0.5	0.01
AT3G01190	Peroxidase 27	-0.6	-0.1	0.009
AT3G32980	Peroxidase 32	-0.5	0.1	0.004
AT4G21960	Peroxidase 42	-0.6	0.1	0.02
AT4G30170	Peroxidase 45	-0.6	0.06	0.004
AT5G64100	Peroxidase 69	-1.1	-0.3	0.003
AT5G64110	Peroxidase 70	-1.0	-0.4	0.0005
AT5G64120	Peroxidase 71	-0.6	-0.1	0.005
**Cluster 3 genes**				
AT1G48130	1-cys-Peroxiredoxin	-0.1	1.7	0.009
AT2G01270	(QSOX2) thioredoxin domain containing protein	0.4	1.1	0.001
AT4G04930	DES-1-like transmembrane protein	-0.09	1.4	0.01
AT2G01180	Phosphatidate phosphatase	0.1	1.0	0.001
AT2G27810	Nucleobase-ascorbate transporter 12 (NAT12)	-0.07	1.6	0.009
AT2G01170	CAT3	0.45	1.2	0.0002

Representative oxidative stress-related genes from Clusters 1, 2, and 3.

Table S7 Growth of wild-type and CRES-T plants in the vertical split media assays shown in Fig. S7

Line	Control		PQ (5 nM)	
	L	R	L (-PQ)	R (+PQ)
Fresh weight (mg)				
WT	5.7 ± 0.7[a]	5.9 ± 0.7[a]	7.6 ± 0.8[b]	0.5 ± 0.1[c]
CRES-T	5.5 ± 0.6[a]	5.8 ± 0.6[a]	5.1 ± 0.6[d]	1.6 ± 0.2[e]
Root length (mm)				
WT	146 ± 5.3[a]	140 ± 5.6[a]	117 ± 5.8[b]	9.4 ± 5.7[c]
CRES-T	157 ± 8.7[a]	140.3 ± 3.1 [a]	136 ± 6.6[d]	41 ± 8.2[e]

Absolute values (mean ± SE) of root fresh weight and the longest root length measured from the split root assay in Fig. S7 are presented. In heterogeneous split medium, root biomass of wild-type plants in the side lacking PQ (red) was significantly increased compared to the counterpart in control split medium, and the root biomass increase is likely due to the development of more roots, because root length did not increase. By contrast, CRES-T lines failed to exhibit such an avoidance response. Different characters in the superscript indicate significant difference between genotype and two sides of the medium analyzed by Tukey's test, $P \leq 0.01$.

Table S8 Growth of wild-type and CRES-T plants in the vertical split media assays shown in Fig. 6

Line	Control		Cu		Zn		TPEN		NaCl	
	L	R	L (-Cu)	R (+Cu)	L (-Zn)	R (+Zn)	L (-TPEN)	R (+TPEN)	L (-NaCl)	R (+NaCl)
Fresh weight (mg)										
WT	1.9 ± 0.1[a]	1.8 ± 0.1[a]	2.8 ± 0.1 [b]	0.5 ± 0.1[c]	2.9 ± 0.3[b]	1.6 ± 0.1[d]	2.6 ± 0.2 [b]	1.5 ± 0.3[d]	3.0 ± 0.1[b]	1.3 ± 0.2[d]
CRES-T	2.2 ± 0.1[a]	1.9 ± 0.1[a]	1.9 ± 0.1[a]	1.9 ± 0.2[a]	2.0 ± 0.3[a]	2.1 ± 0.2[a]	2.4 ± 0.3[a]	2.3 ± 0.1[a]	4.5 ± 0.6[e]	1.3 ± 0.3[d]
Root length (mm)										
WT	165 ± 13[a]	151 ± 14[a]	133 ± 14 [b]	19 ± 3.8[c]	147 ± 8[e]	95 ± 3.6[f]	149 ± 5.8 [a]	109 ± 9.7[f]	164 ± 6.4 [a]	72 ± 9.1[h]
CRES-T	173 ± 21[a]	150 ±7.9[a]	132 ± 7.5[b]	120 ± 6.1[d]	124 ±8.1[d]	104 ± 4[g]	133 ± 7.3[b]	138 ± 9.9[b]	146 ± 9.9 [e]	68 ± 11[h]

Absolute values (mean ± SE) of root fresh weight and the longest root length measured from the split root assay in Fig. 6. In heterogeneous split media, root biomass values of wild-type plants in sides with supplementation with Cu, Zn, TPEN, and NaCl (blue) significantly decreased, whereas those in sides with supplementations (red) significantly increased, compared to the counterparts in control split media. Such root biomass increases in –Cu, -Zn, -TPEN, and –NaCl sides are likely due to development of more roots, and not to increase of root length. By contrast, CRES-T lines exhibited an obvious root biomass increase only in the side lacking NaCl. Different characters in the superscript indicate significant difference between genotype and two sides of the medium analyzed by Tukey's test, $P \leq 0.01$.

Method S1 Analysis of transcriptional activation activity and promoter binding assay.

Transcriptional activation activity of GPL4 was tested in two different ways. First, it was examined using a transient expression assay in Arabidopsis rosette leaves as described previously (Hiratsu *et al*., 2002). The effector (GPL4-CDS fused to the GAL4 DB domain), reporter (GAL4-LUC), and reference (luciferase from *Renilla*) plasmids were delivered into the rosette leaves of 4-wk-old *Arabidopsis* plants by particle bombardment (BIORAD). Bombarded leaves were incubated for 12 h in darkness, and then luciferase activity was quantified using a Dual Luciferase Kit (Promega, Madison, Wisconsin, USA). For the promoter binding assay, Arabidopsis leaves were transformed with the effector (GPL4-CDS), reporter (promoter of candidate genes fused to the CDS of luciferase), and reference (luciferase from *Renilla*) plasmids, and luciferase activity was measured as above using a Tecan spectrophotometer (Infinite M200PRO).

Secondly, GPL4 transcriptional activation activity was tested in a yeast system. The entire CDS of *GPL4* was cloned in a pPC62 vector in fusion with the LexA DNA-binding domain. The construct was introduced into yeast strain L40 (MATα, *his3* -Δ200, *trp1* *leu2-3112, ade2, LYS2::[LexAop(x4)-HIS3], URA3::LexAop[x8]-LacZ, GAL4*). Yeast transformation and the X-gal assay of transformed yeast cells were performed as described (Lee *et al*., 2009).

Method S2 Yeast one-hybrid (Y1H) analysis.

A modified MATCHMAKER One-Hybrid System (Clontech Laboratories, Mountain View, CA, USA) was used to perform the Y1H to analyze the direct binding of promoters of selected genes and GPL4. pHisi-1 vectors harboring the promoters (1.5–2 kb) of each selected candidate gene were integrated into the genome of yeast strain YM4271, and transgenic strains were selected on synthetic dextrose medium agar plates without Ura and His (SD/-UH). To further verify the insertion of the promoters in the genome, yeast gDNA was extracted and PCR was performed with primers specific to the promoter region of each gene (Table S1). The transformed yeast cells harboring the target promoter were transformed again with a pGAD424 vector containing GPL4, and double transformed yeast cells were selected on synthetic dextrose medium lacking Ura and Leu (SD/-UL). The interaction between GPL4 and the target promoters was assessed by growth on SD medium lacking Ura, Leu, and His (SD/-UHL), but supplemented with 30 mM 3-amino-1,2,4-triazole (3-AT; Mitsuda *et al*., 2010).

Notes S1 Accession numbers.

Sequence data from this article can be found in the Arabidopsis Genome Initiative database under the following accession numbers: GPL4 (At1g44810), the closest GPL4-Homologue (At4g00250), GPL1 (At2g25650), GPL2 (At5g13280), GPL3 (At2g36340), 2OG-Fe(II)-dependent oxygenase (AT5G54000), QSOX2 (AT2G01270), Trx-m3 (At2g15570), peroxiredoxin (AT1G48130), CAT3 (AT2G01170), NAT12 (AT2G27810), GST1 (AT5G17220), Zn finger TF (AT3G50700), glyoxal oxidase (AT5G19580), proline oxidase (AT3G30775), MYB75 (AT1G56650), LDOX (AT4G22880), peroxidase 36 precursor (AT3G50990), DES-1-like transmembrane protein (AT4G04930), and phosphatidate phosphatase (AT2G01180).

References

Hiratsu K, Ohta M, Matsui, Ohme-Takagi M. 2002. The SUPERMAN protein is an active repressor whose carboxy-terminal repression domain is required for the development of normal flowers. *FEBS Lett*. **514:**351-354.

Lu D, Wang T, Persson S, Mueller-Roeber B and Schippers JHM. 2013. Transcriptional control of ROS homeostasis by KUODA1 regulates cell expansion during leaf development. *Nat. Commun*. **5:** 3767.

Mitsuda N, Ikeda M, Takada S, Takiguchi Y, Kondou Y, Yoshizumi T, Fujita M, Shinozaki K, Matsui M, and Ohme-Takagi M *et al.* 2010. Efficient yeast one-/two-hybrid screening using a library composed only of transcription factors. *Plant Cell Physiology* **51:** 2145–2151.
